# Hierarchical Artificial Muscle with Nonlinear Elasticity for Antagonistic and Cyclic Robotics

**DOI:** 10.1002/advs.202521604

**Published:** 2026-03-19

**Authors:** Samuel Tsai, Liuyang Cheng, Ali Albazroun, Qiong Wang, Jeongmin Kim, Arman Tekinalp, Soonwook Kim, Charlie Simcox, Ryne Downing, Vagish Sivaramakrishnan, Grace Carsello, Miles Bimrose, Wonsik Eom, William P. King, Mattia Gazzola, Sameh Tawfick

**Affiliations:** ^1^ Department of Mechanical Science and Engineering Grainger College of Engineering University of Illinois Urbana‐Champaign Urbana USA

**Keywords:** coiled artificial muscle, soft actuator, soft robotics

## Abstract

A key design motif of skeletal muscles is their arrangement in pairs to enable the cyclic, contra‐lateral contractions necessary for motion. This mechanism may initially appear inefficient, since the contraction of a muscle group stretches the antagonist, increasing resistance and energy consumption. However, the hierarchical architecture of muscles provides a clever solution. By giving rise to J‐shaped stress–strain responses, muscle tissue is soft at small strains, thus minimizing resistance, while it stiffens at large strains to enable economical energy release and prevent excessive elongation and damage. Here, we develop hierarchical supercoiled artificial muscles by plying fishing line fibers that recapitulate this behavior and thus allow antagonistic actuation. Computational models based on Cosserat rods reveal the physical mechanisms underlying the observed J‐shaped responses. The artificial muscles are used in an antagonistic biceps/triceps arm mechanism and a vertical rope‐climbing robot that weighs 14.4 grams and carries a payload 14.6 times heavier than its own weight.

## Graphical abstract

We construct hierarchical muscles by plying nylon fibers around a heating wire. The hierarchical muscle shows a J‐shaped passive curve, which shows benefit in antagonistic muscle pair and work accumulation mechanism. We also develop a computational, first‐principle model to understand the physics of both active actuation stroke and passive J‐curve.

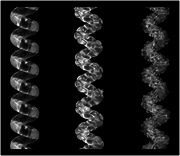

## 1 Introduction

Animal locomotion relies on the biomaterial intelligence encoded in the muscles. Most natural muscles are arranged in groups and pairs to accomplish not only complex motions but also complex work cycles [1]. Locomotion relies on precisely timed alternating contractions of agonist and antagonist muscle groups, as has been demonstrated by in vivo measurements of neural activities in animals [2]. But this cyclic motion initially appears inefficient as the agonist muscle must stretch the antagonist to produce motion. Consider this extreme hypothetical situation: if the active force of the agonist muscle is equal to the passive force of the antagonist due to stretching, the net work from the pair will be zero, and the passive muscle will function as a brake. Hence, the muscles must provide a much higher active force when stimulated than their passive stretching force, which has been the central discovery of the seminal work of Hill in the 1920's [3]. These biomechanics rules for efficient and agile locomotion motivated us to explore of the intricate nonlinear mechanics of artificial muscles and their principles of use in antagonistic arrangements and periodic work cycles.

Natural muscles exhibit a nonlinear J‐shaped stress–strain curve as illustrated by the pioneering experiments of Hill in the 1920s [4]. This nonlinearity makes muscles soft at low stretching loads, sometimes referred to as the toe region, and considerably stiffer at higher loads, after the inflection referred to as the heel region. This behavior prevents joints dislocations or other soft tissue damage at high strains, and it is further believed to favor elastic energy storage and release in antagonistic muscle arrangements, thus supporting efficient animal locomotion [5].

To the best of our knowledge, bioinspired synthetic muscles possessing J‐shaped nonlinear elasticity and their use in soft robotic mechanisms –which exploit their nonlinearity– have never been previously the focus of studies in the literature nor systematically benchmarked in these use cases to measure the performance enhancements that they offer. Many compliant actuators possess hierarchical organizations akin to biological muscles. In particular, coiled artificial muscles are contractile actuators where the structural hierarchy can be encoded through multifilament arrangements made with carbon nanotubes [6, 7, 8, 9], polymers [10, 11, 12, 13, 14, 15, 16, 17, 18, 19], or shape memory alloys [20]. Notably, Aziz et al. show the passive response of a J‐shaped curve when the strain exceeds 200% [18] –the classical geometric nonlinearity expected due to the coiled geometry that straightens at large strains. Aziz et al. do not discuss the behavior of the J‐shaped curve at strains below 30%, and the plots do not appear nonlinear at these small strains. This is a subtle but important difference from the nonlinearity of the toe region of the biological muscles. The nonlinearity of natural soft tissue, muscles, tendons, and ligaments is apparent from the onset of loading, and the inflection point, denoted as the toe region, is at 30% [21], after which the slope changes drastically to prevent overstretching. For natural soft tissue, the nonlinear elasticity from 0% to 30% strain is critical for operation. This nonlinear behavior within a small strain range cannot be accomplished by the geometric nonlinearity of stretching a coil. In this study, we focus on the nonlinear stress‐strain below 30%; and we investigate the underlying mechanism responsible for the J‐shaped stress–strain response, beyond the coil straightening‐induced nonlinearity. Furthermore, we systematically study and benchmark the advantages of such J‐shaped behavior in robotic systems.

**FIGURE 1:**
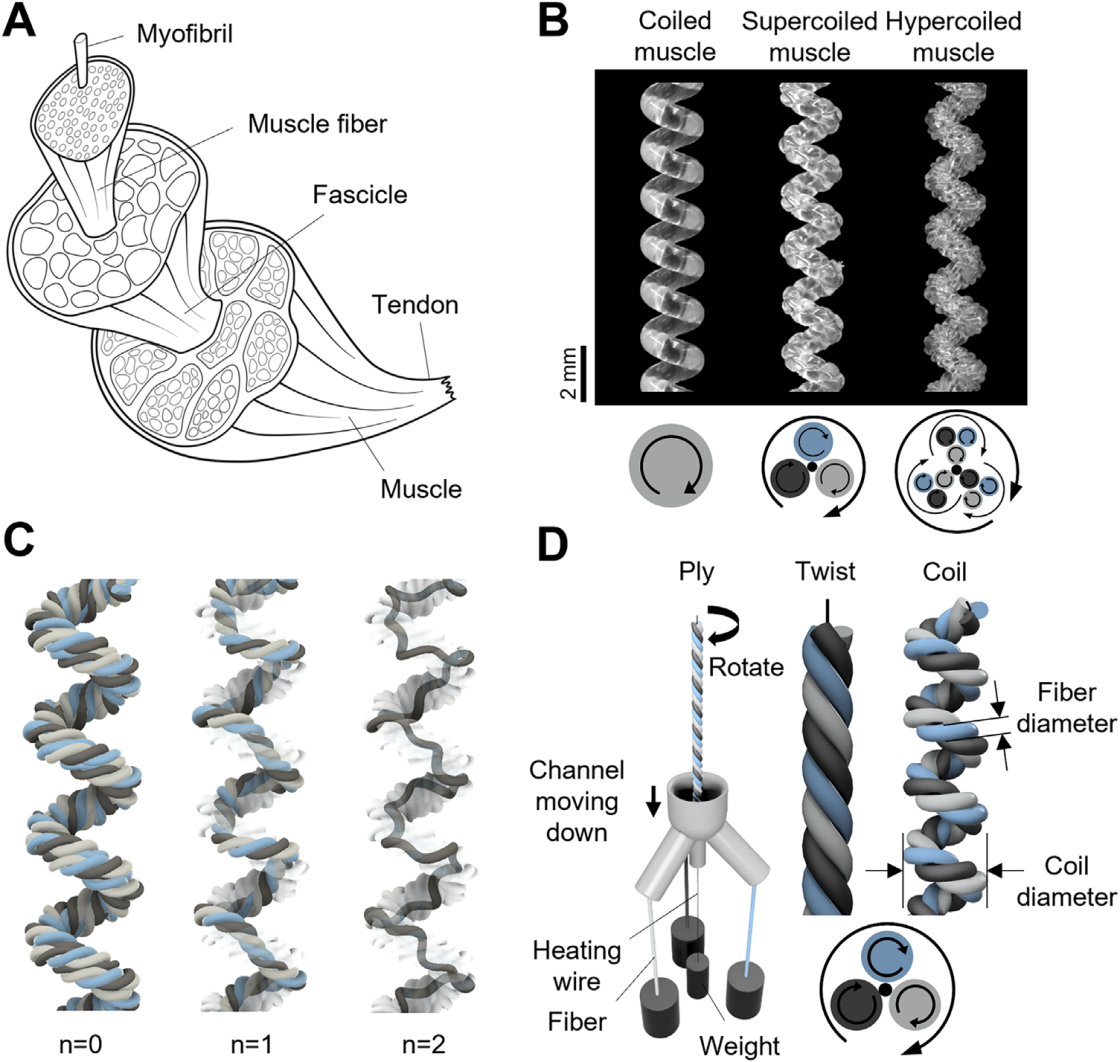
Bio‐inspired hierarchical structure of super‐ and hypercoiled muscles. (A) Schematic of the hierarchical structure found in human skeletal muscle. (B) Comparison of coiled muscle (one fiber), supercoiled muscle (three fibers and one heating wire), and hypercoiled muscle (nine fibers and one heating wire). (C) Hierarchical structure of hypercoiled muscle. Letter 'n' is the hierarchy level. (D) Supercoiled muscle fabrication process schematic. The first step involves plying three nylon fibers (0.47 mm) and one nichrome heating wire (0.1 mm) together. The fibers and the wire are secured at the top end and passed through the branches of the channel. Weights are attached to the lower ends of the fibers and wire. During the plying process, the channel moves downward while the top motor rotates. The second step includes twisting and coiling the three‐ply fiber around a metal mandrel to create a spring‐like geometry, with the final shape being fixed by annealing. The complete fabrication process is shown in the Video S1.

A supercoiled muscle is a multi‐filament coiled actuator inspired by the structural hierarchy of natural muscles. Skeletal muscles are comprised of different length scales ranging from micrometers to meters, as shown in Figure 1A. Supercoiled muscles realize structural hierarchy through the use of three nylon fibers plied around the central nichrome heating wire and then coiled around a metal mandrel (Figure 1B–D). This design establishes a first level of hierarchy (n = 1) relative to standard coiled muscles (n = 0). The coiled muscles (here simply referred to as coiled muscles) are made of a single, twisted nylon fiber. Hypercoiled muscles exhibit a second level of hierarchy (n = 2) by considering nine monofilaments plied together around the central nichrome heating wire (Figure 1B). Specifically, the nine fibers are first separated into three sets, each made of a three‐ply fiber. We then ply these three sets to form the hypercoiled muscle. The nomenclature of super‐ and hypercoiled follows the mathematical terminology of ordered hierarchies [22, 23]. The hierarchy levels are defined from Lakes [24]. Figure 1B and Table 1 compare these three types of muscles.

**TABLE 1:** Comparison of three muscle parameters listed in Figure 1. The fiber diameters are controlled to maintain a similar cross‐sectional area and spring index. The cross‐sectional area of a supercoiled muscle is three times that of a fiber with a diameter of 0.47 mm because the material is incompressible. The cross‐sectional area of a hypercoiled muscle is nine times that of a fiber with a diameter of 0.26 mm. Both supercoiled and hypercoiled muscles use the same heating wire (0.1 mm diameter). The stress is calculated by dividing the force by the total area of the fiber. Of course, this is a nominal engineering stress, used here only as a proxy, which follows all articles published in this area. The true stress state on the material level is a complex deviatoric and hydrostatic stress, but it is not used in this work.

	Coiled	Supercoiled	Hypercoiled
Number of fiber(s)	1	3	9
Fiber diameter (mm)	0.80	0.47	0.26
Cross‐section (${\mathrm{mm}}^{2}$)	0.50	0.52	0.48
Coil diameter (mm)	2.43	2.48	2.42
Spring index	1.45	1.32	1.30
Embedded heating wire	No	Yes	Yes

We systematically investigate the structural hierarchy of this class of actuators to replicate the J‐shaped stress–strain behavior of natural muscles and identify the microstructural features responsible for this nonlinearity. Hierarchically coiled, thermally actuated artificial muscles are fabricated here by plying various numbers of monofilament nylon fishing lines around a thin heating wire. By controlling the number of fibers and their architecture, we show that hierarchical muscles produce consistent, predictable, and repeatable passive nonlinear J‐shaped elastic responses. We show using experiments infrared imaging and finite element thermal modeling that the repeatability of this response is facilitated by uniform temperature distribution. Using a Cosserat rod model, we analyze the various material, frictional, and geometric properties, which drive the passive and total stress performance of these hierarchical structures. This model has the ability to predict nonlinear performance from basic material properties. Finally, we demonstrate that, compared to conventional coiled muscles, hierarchical muscles achieve greater work capacity even at comparable stroke, but more importantly, they possess higher passive strain to active stroke ratio. Owing to these mechanical and thermal enhancements, when integrated into antagonistic robotic arm mechanisms and work‐accumulation systems, hierarchical muscles improve performance by 75% and 480%, respectively.

### 1.1 Active and Passive Performance of Super‐ and Hypercoiled Muscles

**FIGURE 2:**
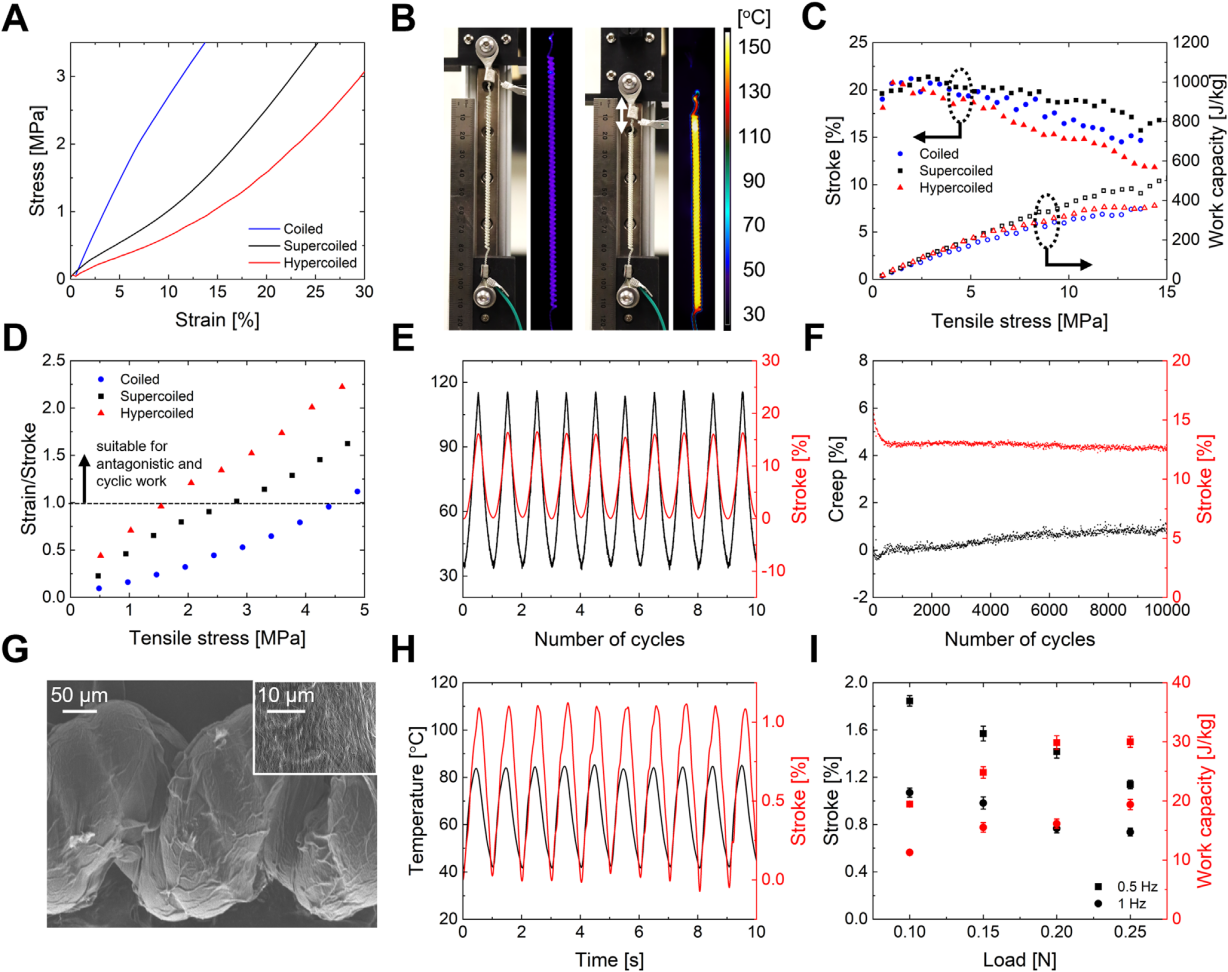
Coiled, super‐, and hypercoiled muscle stress‐strain curve and actuation performance (A) Tensile test results of the three muscles. (B) Optical and infrared images of the resting (left) and actuated (right) supercoiled muscles. The initial length is 80 mm, and the resistance of the heating wire is 41.6 Ohms. The supercoiled muscle shows uniform temperature during actuation. More detailed comparison can be found in Figure 3. Videos are available in Video S2. (C) Isobaric test and work capacity of the three muscles. (D) Ratio of passive strain and actuation stroke of the three muscles. (E) The first ten cycles of temperature and stroke for the 10000‐cycle test from supercoiled muscle. A 1 N force is applied at the end of the muscle, and the actuation frequency is 0.1 Hz with 50% duty cycle. (F) Supercoiled muscle creep and stroke of the 10000‐cycle test, with each data point representing an average of ten measurements. Note that all stresses are normalized by the fiber cross‐sectional area. Strain and stroke are normalized by the muscle initial unloaded length. (G) SEM images of supercoiled muscle made with 50 μ m nylon fibers and coated with carbon nanotube sheath for heating. (H) Temperature and stroke at 1 Hz under 0.1 N of the supercoiled muscle shown in (G). (I) The stroke and work capacity versus load from 0.1 to 0.25 N at 0.5 and 1 Hz.

Figure 2A compares the passive stress–strain response of the three muscles. Coiled muscles exhibit an almost linear stress–strain response in the investigated strain range. In contrast, both supercoiled and hypercoiled muscles show a nonlinear stiffening stress–strain response, reminiscent of biological muscles. The elastic nonlinearity is J‐shaped, characterized by relatively low stress at small strains, followed by a nonlinear increase with strain. The passive force‐displacement curve reveals the considerable effect of the structural hierarchy, whereby the hypercoiled muscle is softer and more nonlinear than the supercoiled muscle.

**FIGURE 3:**
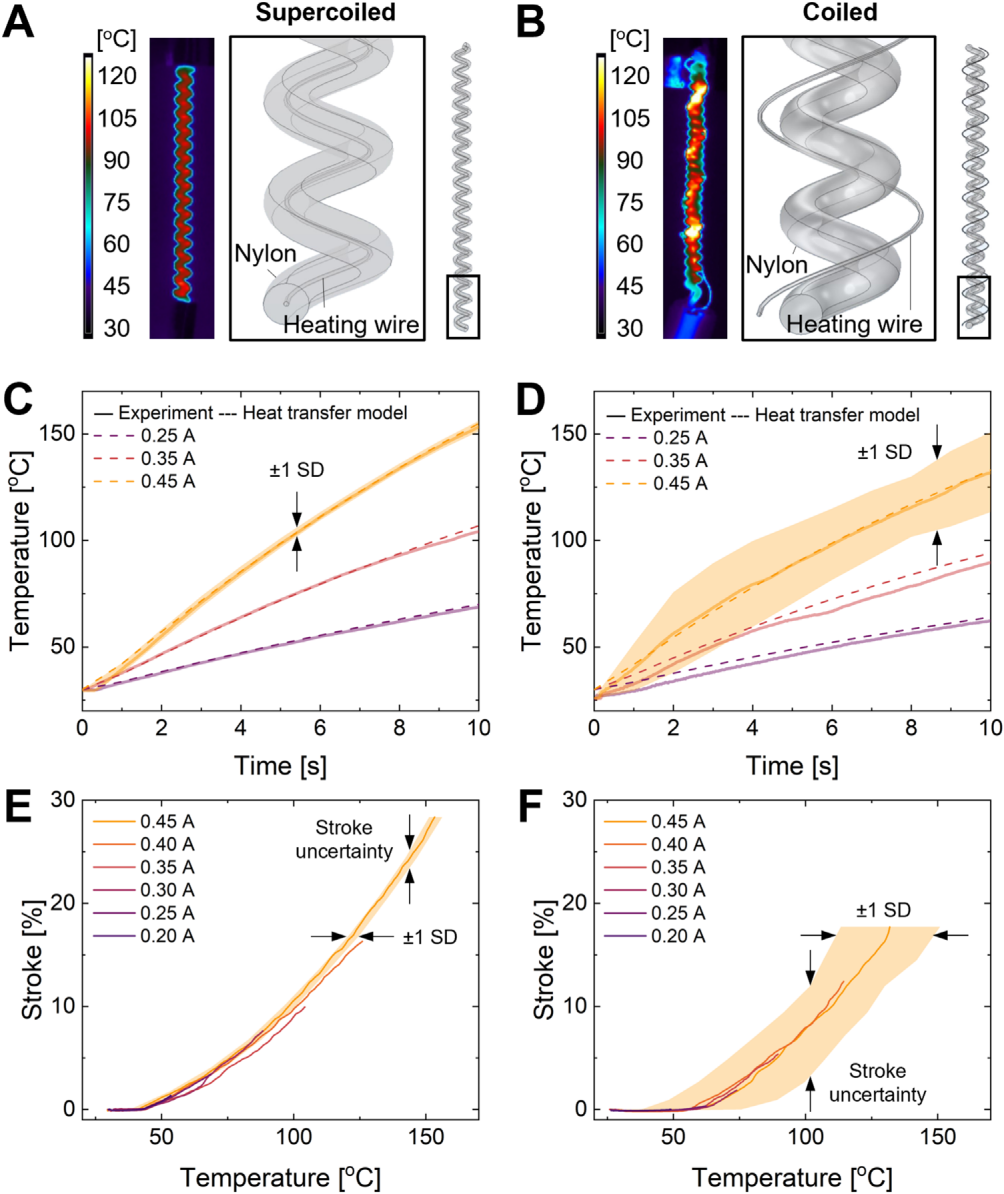
Supercoiled muscle temperature uniformity, heat transfer modeling, and stroke repeatability. (A, B) Infrared images taken during actuation. Both muscle lengths and heating wire lengths are the same. The right side displays the 3D heat transfer model from COMSOL Multiphysics, which reproduces the experiment. Both 3D models have the same polymer volume and heating wire length. The coiled muscle is wrapped with an external heating wire, and there is a small air gap between the heating wire and the nylon to replicate the experimental observation. The supercoiled muscle has an embedded heating wire in the center of the nylon muscle, enabling conduction between the surface of the heating wire and the surrounding nylon. (C) Supercoiled muscle and (D) coiled muscle heat transfer model versus experimental measurements with different current inputs. The mean temperatures of each current are shown in the solid lines. The shaded regions show the standard deviation of the temperature range measured using infrared images along each muscle with a current of 0.45 A. (E) Supercoiled muscle and (F) coiled muscle temperature versus stroke with different currents, illustrating the temperature uniformity and stroke consistency of supercoiled muscle.

The muscle actuation performance (Figure 2B,C) is evaluated through the isobaric (constant stress) test. Figure 2B presents the optical and infrared images of a passively stretched and actively heated (thus contracting) supercoiled muscle. Note the uniformity of temperature due to the internally embedded heating wire compared to the hot spots obtained in coiled muscles from Figure 3B. We note that many previous experiments that report uniform temperature distribution in coiled muscles use a heating gun or an external heated environment. It is practically challenging to realize such uniform temperature distribution with a heated wire due to localization of hot spots. Figure 2C compares the strokes and work capacity assessed within the temperature range of 30 to 150 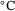. In the small stress region, all three muscles follow similar trends and perform comparably, with a peak stroke observed at 2–3 MPa, due to the fact that at small loads, coil–coil contact prevents the muscle from contracting further. As the applied stress increases, the strokes generally decrease, and the supercoiled muscle maintains larger strokes than hypercoiled muscles. The stroke differences among the three muscles are minimal, whereas the passive response differences are substantial.

Since the stroke varies with the applied load for contractile actuators, we compare the muscles in terms of their work capacity in Figure 2C. Work capacity is defined by the work generated by the muscle (hanging weight multiplied by vertical displacement) divided by the total mass of the muscle, which includes the internal heating wire. It is worth noting, for reference, that the heating wire accounts for only 10% of the total mass of the supercoiled muscle. Instead, for coiled muscles, the heating wire wrapped around the nylon filament contributes as much as 37% to the total mass, which increases overall volume and causes thermal hot spots, as illustrated in Figure S1. As a result, the supercoiled muscle exhibits higher work capacity and reduced size compared to the coiled muscle. Similar considerations apply to hypercoiled muscles, which are indeed found to perform similar work capacity as the coiled muscles. Overall, the results indicate that hierarchical muscles exhibit considerable non‐linear J‐shaped stress–strain behaviors, and a small improvement in the work capacity. This leads to much higher strain to stroke ratios, as shown in Figure 2D. The stroke differences among the three muscles are minimal, whereas the passive response differences are substantial. This changes the strain to stroke ratio reaching values larger than one for supercoiled and hypercoiled muscles. A large strain‐to‐stroke value of the muscle indicates that the muscle is passively stretchable, which makes it suitable for an antagonistic arrangement, as will be described later in this article. However, since supercoiled muscles achieve a higher ratio of passive strain to active stroke compared to coiled muscles, namely larger than one, while having simpler manufacturing compared to the hypercoiled muscles, the remainder of this paper will only focus on this architecture. We note that hypercoiled muscles indeed have a higher passive strain to active stroke ratio, which might be suitable for some applications.

Figure 2E shows the results of the isobaric test of supercoiled muscles for the initial ten cycles in a series of 10,000‐cycle tests, conducted at a frequency of 0.1 Hz. The weight attached to the end of the muscle is 1 N, resulting in a nominal stress of 1.9 MPa. A 0.52 A current is supplied with 50% duty cycle. The stroke is around 15% and the temperature ranges from 35 to 115 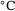. Figure 2F instead presents the full results of the 10,000‐cycle tests. Notably, the stroke stabilizes after the initial 500 cycles and maintains a constant value of 12.5% throughout the entire test duration. Remarkably, the supercoiled muscle shows minimal length change even after a long testing period. The length change is described as 1% creep, which is defined as the muscle rest (unactuated) length divided by the initial loaded length. Hypercoiled muscle cyclic test results are shown in Figure S2. The results indicate that the hierarchical muscle is capable of delivering repeatable strokes with minimal deformation over extended testing cycles [25]. Notably, the fatigue and cyclic properties measured for supercoiled muscles are steady and comparable to coiled muscles despite the complex architecture of these muscles and the presence of various internal components, such as three fibers and a metal wire, which could in principle slide and cause hysteresis.

We also demonstrate a high‐actuation‐frequency supercoiled muscle by scaling down the nylon fiber diameter to 50 μ m, as shown in Figure 2G,H,I. Figure 2G shows the nylon supercoiled muscle coated with CNTs for Joule heating. CNT coating on the outer surface of the supercoiled muscle is more suitable for imparting electrical conductivity to these miniature muscles compared to embedding a heating wire in the inner core of the ply because the large plying and twisting stresses break thin metal wires of diameter less than 20 μ m. Figure 2H plots the temperature and stroke at 1 Hz actuation frequency. The displacement peaks and valleys align with the corresponding temperature peaks, indicating that the muscle is highly responsive to temperature, owing to its small diameter and, hence, its small heat capacity. Figure 2I compares the stroke and work capacity of the muscle at 0.5 and 1 Hz, for different loading conditions but maintaining the same maximum voltage of 29 V. Beyond this voltage, the CNTs used for heating degrade. At 0.5 Hz, the muscle reaches a higher stroke of 1.8% versus 1.1% at 1 Hz due to the higher heating energy at 0.5 Hz. Similarly, the temperature range for 1 Hz is from 40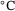 to 95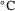 (Figure S3C), and the temperature range for 0.5 Hz is from 30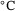 to 105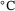 (Figure S3E). We note that for any electrothermal actuator, the stroke decreases with actuation frequency until it eventually vanishes at a certain frequency. Frequency‐dependent stroke behavior in twisted nylon muscles has been previously reported [26]. Similar characteristics have also been observed in other types of soft actuators, including dielectric elastomer actuators [27, 28, 29] and liquid crystal elastomers [30], indicating that this behavior is a general feature of soft actuation systems. It is a noteworthy performance to achieve 1 Hz actuation electrothermally at this scale, and still maintain a stroke of 1%. The high‐frequency muscles show a high work capacity of 30 J kg^−1^, comparable to biological muscles, owing to their light weight of 0.095 mg mm^−1^.

### 1.2 Temperature Uniformity and Stroke Effectiveness

Electrical stimulation of supercoiled muscles with embedded heating wire presents practical benefits in terms of temperature uniformity and stroke effectiveness relative to coiled muscles. We investigated the internal morphology of supercoiled muscles and their benefits on heat transfer. X‐ray tomography scanning shows that the heating wire is in the core of the three‐ply nylon fibers, which in turn deform their polymeric cross‐section to conform to the stiffer surface of the metal electrical wire, as shown in Figure S4. Encouraged by this ideal morphology, where the heat is transferred from the core of the muscle radially outwards to the fibers, we investigated the thermal gradient within the cross‐section. We calculated the Biot number, a dimensionless parameter comparing internal thermal conduction with surface heat transfer in a solid, to be 0.05<<1 for supercoiled muscle, which confirms the temperature uniformity across the diameter of the muscle. Next, we investigated the temperature uniformity along the length of the muscle. Figure 3A,B compare the coiled and supercoiled muscles under the same energy input. The supercoiled muscle demonstrates higher temperature uniformity along its length, while the coiled muscle exhibits heat concentration, resulting in hot spots near the heating wire. Figure 3C,D compare the mean temperature and standard deviation of both muscles by varying the current input from 0.25 to 0.45 A. The results show that the temperature of the supercoiled muscle has a standard deviation of 2.5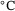, much smaller than the 18.7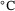 for the coiled muscle.

The temperature uniformity of supercoiled muscles enables predictive heat transfer models in COMSOL Multiphysics. To validate the model, we compare the model with temperature measurements obtained via infrared images. The results from Figure 3C,D indicate that the model accurately predicts the muscle temperature with low uncertainty in the case of supercoiled muscles. Figure 3E,F compare the muscle temperature and stroke. For the same amount of energy input, the supercoiled muscle heats up faster than the coiled muscle due to the internally embedded heat source location and the small Biot number, which transfers thermal energy to the muscle more efficiently, minimizing heat loss to the surrounding air. As a result, supercoiled muscles achieve higher temperatures and at a faster rate, leading to increased muscle stroke. The heat transfer model of the supercoiled muscle enables the prediction of muscle temperature and thus the stroke from the input current. This is advantageous for all practical applications of such actuators in engineering systems. Moreover, the model also can readily be used to predict the stroke with new thermal boundary conditions which is again practically crucial when the muscle is inserted or embedded in an engineering system. Finally, the model can be used to optimize the geometry and identifies key design parameters to enhance the stroke.

### 1.3 Computational Modeling of Muscle Dynamics

**FIGURE 4:**
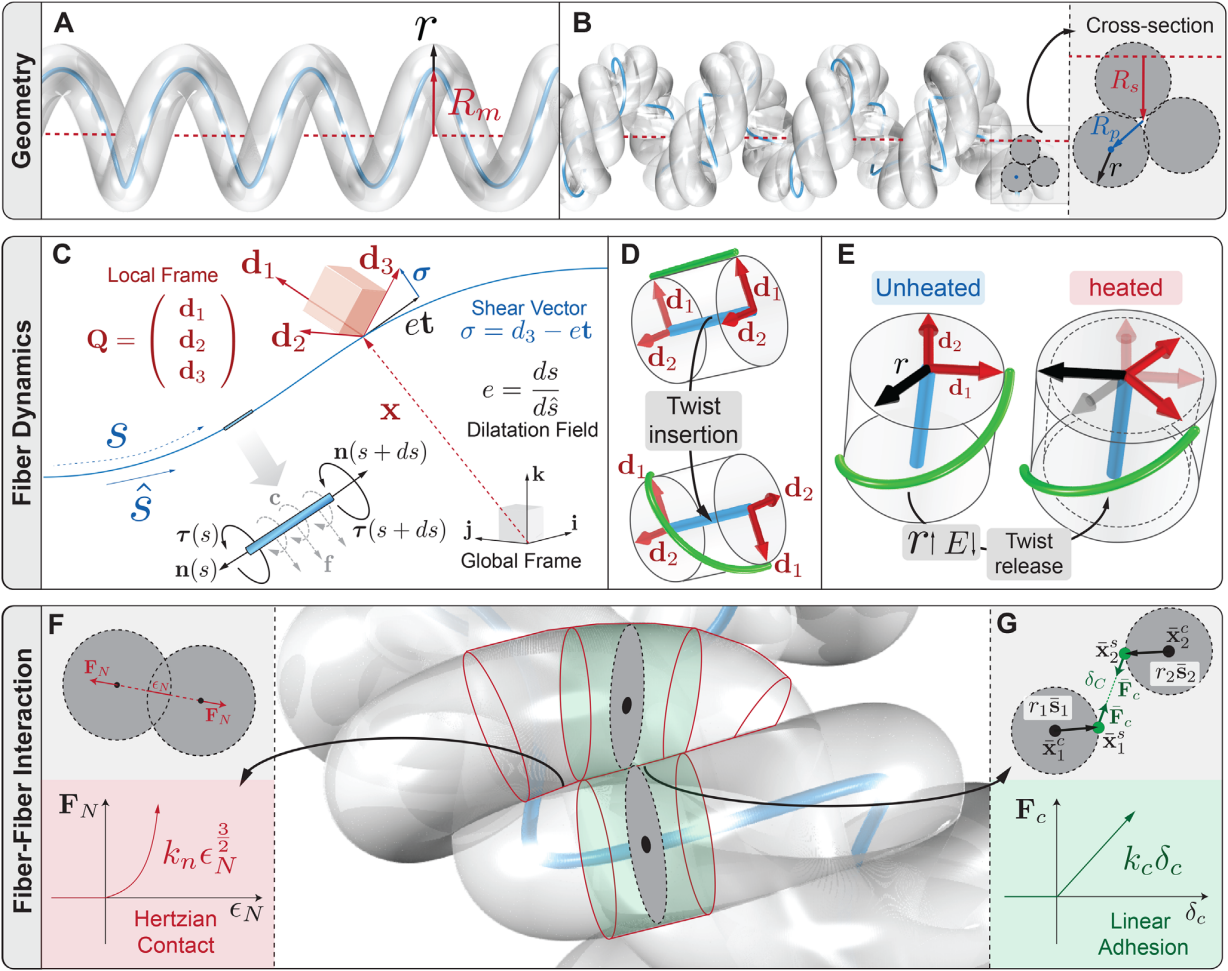
Computational modeling of coiled artificial muscles. (A) The coiled muscle and (B) the supercoiled muscle are parameterized based on the fabricated muscle geometry, see Muscle Geometry Parametrization for details. (C) Muscle fibers are modeled as Cosserat rods, see Cosserat Rod Theory for details. (D) Local frames at consecutive rod elements are rotated with respect to each other to match the twist inserted into the muscle fibers during the manufacturing. (E) Fibers are actuated via internal forces n and torques τ that result from replicating thermal actuation effects such as fiber radial r expansion and decrease in Young's modulus E. (F) A Hertzian contact model is used where the force is proportional to the penetration $\varepsilon_{N}$. For each element, the contact force is applied based on the penetration between this element and the closest three elements in the neighboring fibers (highlighted in red outlines). (G) A linear spring model for adhesion is implemented. The spring is between neighboring surfaces on different fiber elements (highlighted in green). See Adhesion Model for details.

We construct a computational model of the coiled and supercoiled muscles to analyze their dynamic behavior in passive and active states, understand physical origins of the J‐curve, enable stroke predictions from material/geometric properties, and design multi‐muscles mechanisms. Our physically revealing, first‐principles approach, sets our model apart from previous data‐driven ‘supercoiled polymer’ SCP models [11, 31, 32, 81]. We begin with reconstructing the muscle fiber geometry to formulate individual fiber mechanics. Next, we incorporate fiber–fiber interactions to capture the emergent dynamics of the hierarchical structure. The geometry of each muscle fiber is captured using the parametric equations of a helix (coil) and a helix around a helix (supercoil), as presented in the Muscle Geometry Parametrization section of the Materials and Methods. The parameters of the model are chosen to match the geometry of the manufactured muscles as shown in Figure 4A,B. Each fiber is modeled as a Cosserat rod, illustrated in Figure 4C. Cosserat rod theory [33] exploits fiber slenderness to model 3D dynamics and deformations (bending, twisting, shearing, stretching) through a 1D Lagrangian representation. The governing equations are presented in the Cosserat Rod Theory section of the Materials and Methods. We solve the Cosserat rod equations numerically using the open software Elastica [34], whose accuracy and utility in capturing fiber‐based system dynamics has been extensively demonstrated through rigorous benchmarks [35, 36], quantitative simulations of mechanical instabilities [36, 37], animal locomotion [36, 38, 39, 40], plant dynamics [41], fibrous metamaterials [42, 43], and for the design and control of artificial [44, 45, 46, 47, 48, 49], as well as bio‐hybrid [50, 51, 52] soft robots.

The inserted twist per fiber length of the muscle has a significant effect on the muscle tensile stroke [53], and is not captured by only parameterizing the geometry. We thus additionally twist the Cosserat rods as in Figure 4D, to match the twist inserted during manufacturing. We then proceed with modeling the thermal properties of the muscles by using the stress–strain relations of [54], which were specifically derived for the coiled nylon actuators and capture the behavior of an elastic material (nylon) with temperature dependent modulus. See the Muscle Fiber Thermal Actuation Model section of the Materials and Methods for details. Our prior studies show that temperature dependence of the modulus is key to modeling and understanding the behavior of coiled nylon muscles. As the muscle temperature increases, the Young's modulus E decreases (softening) while the fiber radius r increases (thermal expansion). These changes cause the fiber to untwist (Figure 4E). Twist is then converted into writhe (which can be approximately thought of as bending), leading to larger muscle coils, which in turn cause the length of the actuator to contract, producing the stroke [37].

**FIGURE 5:**
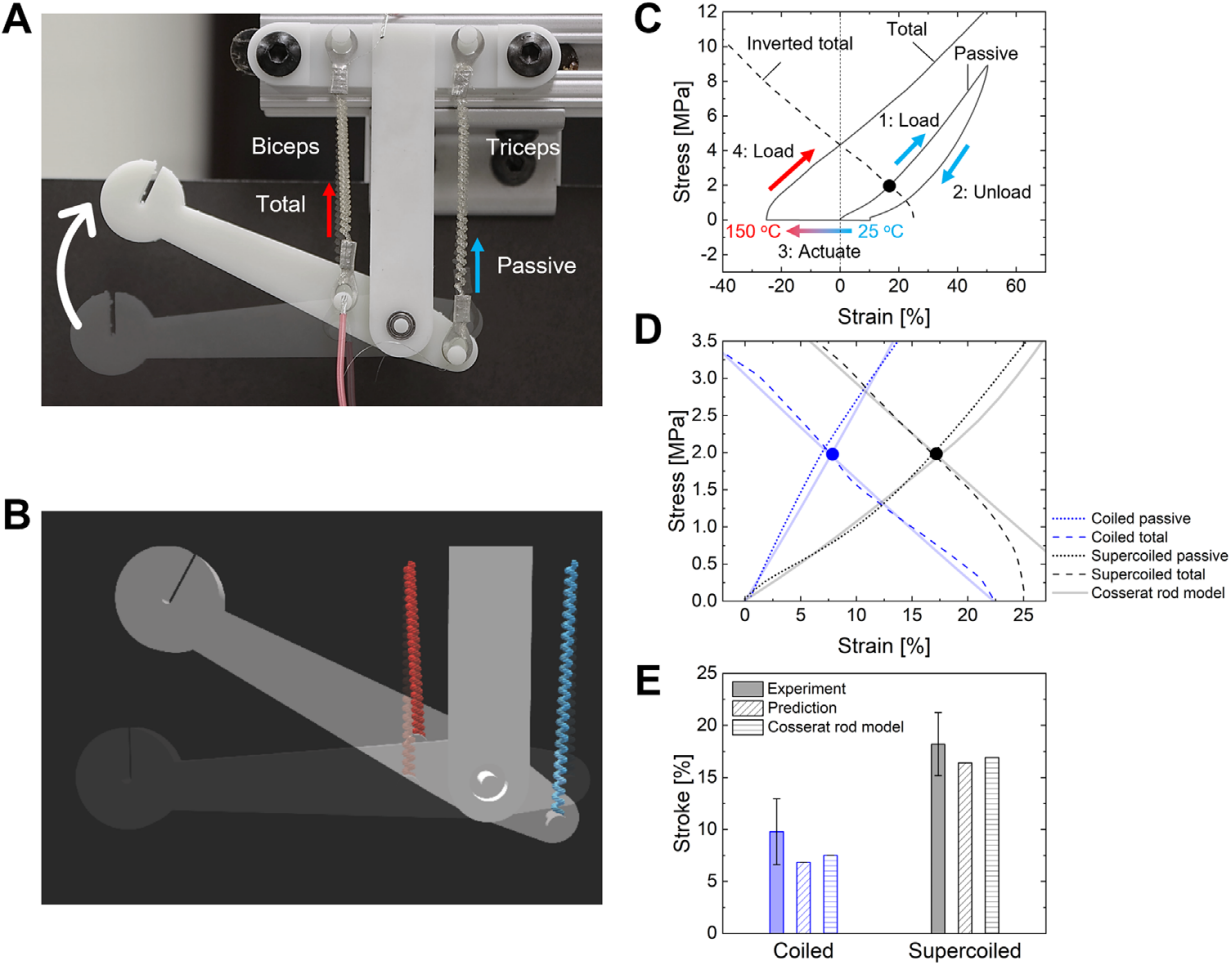
Antagonistic muscle pair experiment and modeling. (A) Experimental setup with supercoiled muscles illustrating the actuation of biceps and extension of triceps. (B) Muscle antagonistic arrangement simulated using PyElastica. (C) Empirical model for the muscle antagonistic test, utilizing passive and total curves obtained from the dynamic mechanical analysis (DMA). The process involves four steps: (1) loading at room temperature, (2) unloading until there is no stress, (3) actuation, where stress is held constant at zero while heating the muscle from room temperature to 150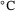, and (4) loading with constant temperature at 150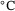. To achieve equilibrium in the antagonistic arrangement, the passive stress and total stress should be equal, and the stroke (negative strain) should match the passive strain (positive strain). Consequently, the total curve is inverted, and the point of intersection between the inverted total curve and the passive curve represents equilibrium. The muscle used in this plot is a supercoiled muscle. (D) Compare the empirical model and Cosserat model of inverted total curves and passive curves. (E) Comparison between the experimental measurements, empirical model, and Cosserat rod model for antagonistic arrangement. Experimental results are measured from (A). Empirical model based on passive and total curves is shown in (C). Cosserat rod modeling is described in Figure 4.

The mechanism described above is sufficient to explain the passive and active dynamic responses of the coiled muscle, as shown in Figure 5D. However, to capture the behavior of supercoiled muscles, the interactions between muscle fibers (contact, sliding, adhesion) need to be considered. We model the contact between the curved surfaces of the muscle fibers using the standard Hertzian contact model [55] of Figure 4F.

We base our models on the hypothesis that the combination of contact forces, adhesion, and sliding among the fibers is critical to producing the non‐linear J‐curve passive response of supercoiled muscles. We performed two controlled experiments to build this hypothesis. In the first experiment, we observe that by separating the fibers of a fully assembled supercoiled muscle and testing their passive behavior in parallel (i.e., without adhesion and sliding), we obtain a linear response, though much softer than the supercoiled muscle as a whole (Figure S5). This response matches the response we get when the fiber interaction effects are ignored in simulation. In the second control experiment, when the fibers in the supercoiled muscle are glued together using epoxy, thus preventing relative sliding, once again a linear response is observed (Figure S6). Similar results are obtained in simulations, whereby contact and/or adhesion, but without sliding, give rise to a linear response of the actuator.

First, we describe the model of the adhesion of the fibers. We employ a linear spring that connects each discretized element along the fiber to the first –closest– neighboring elements of the other fibers, as shown in Figure 4G. Adhesion is captured using a spring $F_{C}$ that connects the surface of one fiber element to the surface of the nearest element in a second fiber. This spring provides a restoring force proportional to the surface‐to‐surface distance $\delta_{c}$ and orientation between the two fiber elements. The spring model is based on [38]. Using this spring alone produces a linear response similar to the one found in experiments with glued fibers, with the spring constant k controlling the adhesion strength.

Next, we model the combined effects of the sliding and the Hertzian contact forces. Again, we first detect the closest neighbor element in the second fiber. Then, we consider the two next neighbors on both sides of the closest neighbor along the second fiber (elements with a red outline Figure 4F). Expanding the contact to these elements allows the calculation of the contact forces, based on the penetration depth, between each element on one fiber and the new neighboring elements in contact with it when the elements slide past each other due to deformation. Summing the contact forces from two neighboring elements finally gives rise to a J‐curve response in the simulated muscles. The parameters involved in the adhesion model (Table S2) are chosen to match the experimental J‐curve of supercoiled muscles, as shown in Figure 5D. Further details can be found in the Materials and Methods Fiber–Fiber Interactions section. Note that this study focuses on strain range <0.3, which is a realistic strain range for robotics application. If the strain is larger than 1, both coiled and supercoiled muscles show a J‐curve, as shown in Figure S7.

### 1.4 Antagonistic Artificial Muscles

Owing to the J‐curve, supercoiled muscles are expected to be well‐suited for implementing antagonistic actuation mechanisms. A classic example is represented by the human arm, which we revisit in Figure 5A by incorporating artificial biceps, triceps, upper arm, and forearm. A digital twin based on Cosserat modeling is instead depicted in Figure 5B. As the biceps actuate, they exert forces that stretch the triceps. Equilibrium is reached when the torque created by the biceps is balanced by the torque created by the triceps. An experimental video is available in Video S3. We used two separate modeling approaches to understand the mechanism of this large enhancement, an empirical model and Cosserat‐based simulations.

The approach for the empirical model is to use isolated muscle testing to predict their antagonistic performance by a quasi‐static force analysis approach. Figure 5C illustrates the force curves measured by dynamic mechanical analysis (DMA 850, TA Instruments). The testing process involves four steps. In the first step, the muscle is subjected to loading at room temperature, which provides a passive force‐displacement curve. The second step involves unloading the muscle to reduce the force to zero. In the third step, the muscle is actuated to 150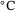 while maintaining zero force. During this step, the muscle contracts, resulting in a negative strain. The final step involves loading the muscle again while maintaining the temperature at 150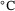. The final step is another force‐displacement measurement in the actuated state. We refer to it as the total force curve because this response combines the passive response with the forces generated from the actuation. The forces are divided by the cross‐sectional area of the supercoiled muscle fibers ‐three fibers for instance‐ to calculate a nominal stress.

Using the passive and total force curves, we construct a empirical model of two muscles, where one is active and the other is in the passive state. To achieve equilibrium in the arm antagonistic arrangement, the torque from biceps and triceps should balance. Since the arm length is the same, the passive force from the triceps muscle should be equal to the total force of the activated biceps, with the strain having the same magnitude but opposite signs. Passive strain has a positive value, while total strain is negative. Consequently, we invert the total force curve to identify the equilibrium position, which is shown by the intersection between the inverted total force curve and the passive force curve. This model captures the observed equilibrium configuration of the arm as shown in Figure 5D, together with the Cosserat simulation. The antagonistic stroke in the case of supercoiled muscles is approximately double that of the coiled muscle. This is due to the lower stiffness of the supercoiled muscle in the small strain region, or the toe region of the J‐curve, the muscle exhibits a larger equilibrium stroke. As can be seen, the two models are found to be quantitatively consistent, confirming the viability of our actuation strategy. Upon experimental implementation (Figure 5E), results indicate that we can accurately predict the final equilibrium configuration of the arm mechanism, thus validating our modeling approaches.

Arm‐mimicking mechanisms are commonly seen in soft actuator applications. However, most of the demonstrations do not include an antagonistic muscle pairs, which also eliminate the ability to perform work cycles [12, 19, 56, 57, 58, 59, 60]. This is because adding an equal antagonistic muscle with linear passive force‐displacement response would significantly reduce the net work, such as lifting the arm against gravity. A notable study uses a complicated muscle pair in actuating an arm mechanism, where both antagonistic muscles are pre‐pressurized in the initial configuration to enable stretchability of the expanding muscle when the contracting muscle pulls the arm upwards [61]. This complication and pre‐pressurization could be eliminated if these pneumatic muscles had a non‐linear passive J‐curve.

### 1.5 Cyclic Work Accumulation Machines

Biological systems accumulate work during each cycle through antagonistic contractions. They achieve this by following a general principle where they operate the muscles cyclically within a force range. For example, a walking animal or a flying bird accumulates locomotion work via cyclic forward and backward strokes of their legs or wings. These forward and backward strokes operate between a larger and smaller force or torque limits. In the context of artificial muscle applications, the net work produced per cycle is crucial for characterizing the muscle when used in a mechanism. Antagonist muscles must maximize the net work per cycle under specific loading conditions and temperature ranges. Supercoiled muscles are particularly advantageous in machines that not only operate with a constant force but also switch between different loads, enabling them to produce a positive net work over repeated actuation cycles.

**FIGURE 6:**
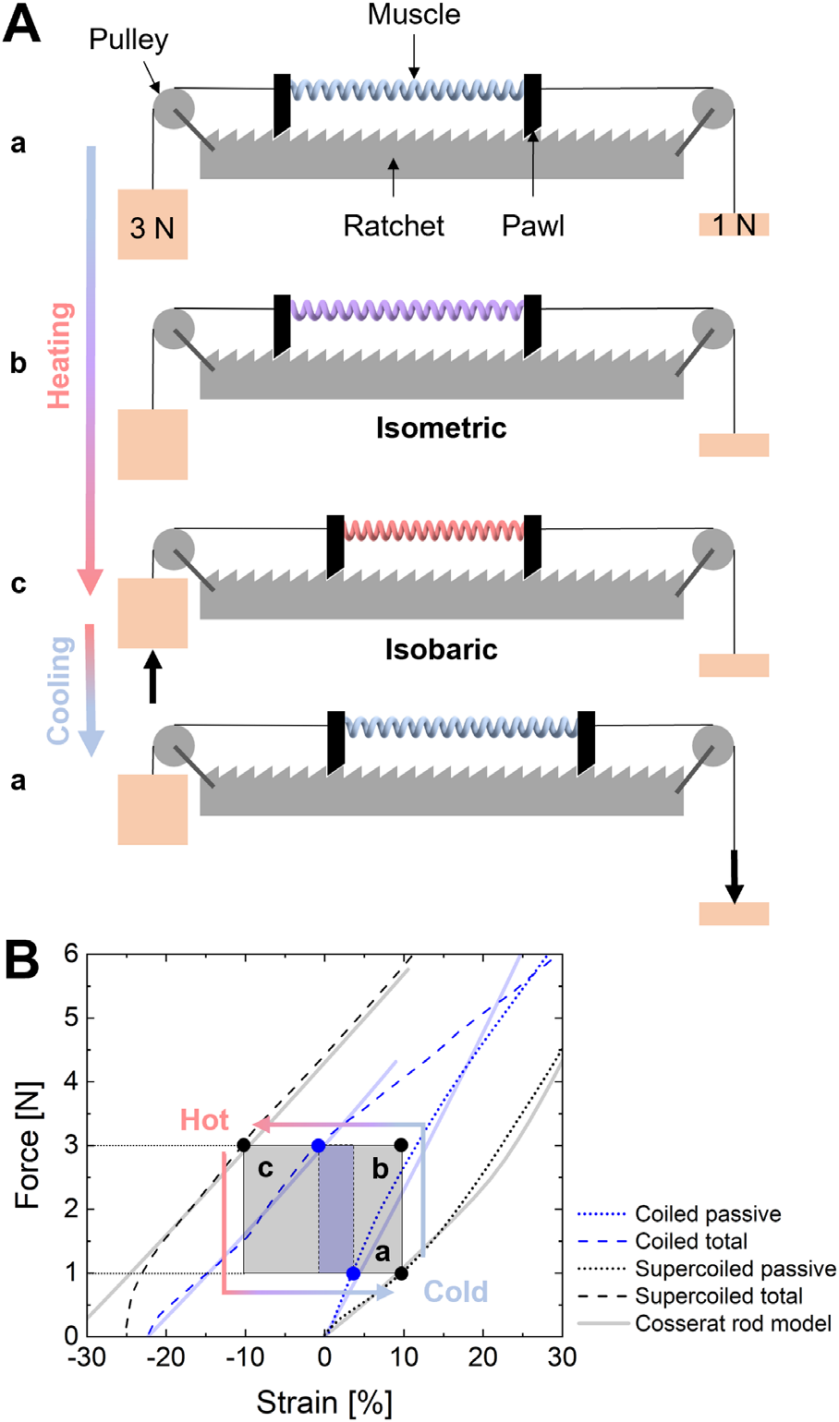
Concept and analysis of the machine accumulating a net work per cycle. (A) Schematic of the work accumulation machine through a complete cycle as it lifts the heavier load while lowering the smaller load. In this machine, the applied force alternates between 1 and 3 N, which corresponds to the weights connected to both sides of the muscle. Each end of the muscle is linked to a pawl, preventing backward motion. From step a to step b, the muscle active force gradually increases as the temperature rises. While the active force is below 3 N, the muscle cannot contract and remains in an isometric state. Once the force exceeds 3 N, the muscle contracts and lifts the 3 N weight, entering an isobaric state from step b to step c. In the cooling process, the muscle active force gradually decreases as the temperature drops. When the active force falls to 1 N, the muscle elongates due to the 1 N weight on the right side stretching the muscle. (B) The area of the rectangle is the net work provided from the muscle to the machine for one complete cycle. Due to the J‐curve and the stronger total force curve, the supercoiled muscle generates more work per cycle for the same force range.

We consider the work accumulating machine illustrated in Figure 6A operating within a force range of 1 to 3 N. The purpose of the machine is to lift the heavier 3 N load and lower the 1 N load. The machine achieves this by cyclic contractions along a one‐way ratchet. The net work produced during each actuation cycle is represented by the area between the total stress curve and the passive stress curve shown in Figure 6B. We note that when the muscle is activated during the forward stroke, it initially undergoes an isometric actuation, increasing the force from 1 N until it reaches the threshold force of 3 N. At this point, the muscle switches from isometric to isobaric, where it lifts the 3 N load. In the backward stroke, the force is first isometrically decreased, followed by the backward isobaric stroke at 1 N. When powered by a supercoiled muscle, the machine accumulates more work per cycle compared to a coiled muscle. This is due to the J‐shaped passive response, which results in lower internal resistance during the muscle restoration phase. Additionally, the supercoiled muscle exhibits a higher total force in the actuated state within the same temperature range, further contributing to the increased work produced per cycle. Figure 6 can be applied to any machine that operates cyclically within a specific force range. The concept of calculating the area between the passive and total stress curves can also be useful in actuator selection. Ideally, the best actuator would have a passive stress curve that is as soft as possible, while the total stress curve in the actuated state should be as steep as possible.

### 1.6 Rope‐Climbing Robot

**FIGURE 7:**
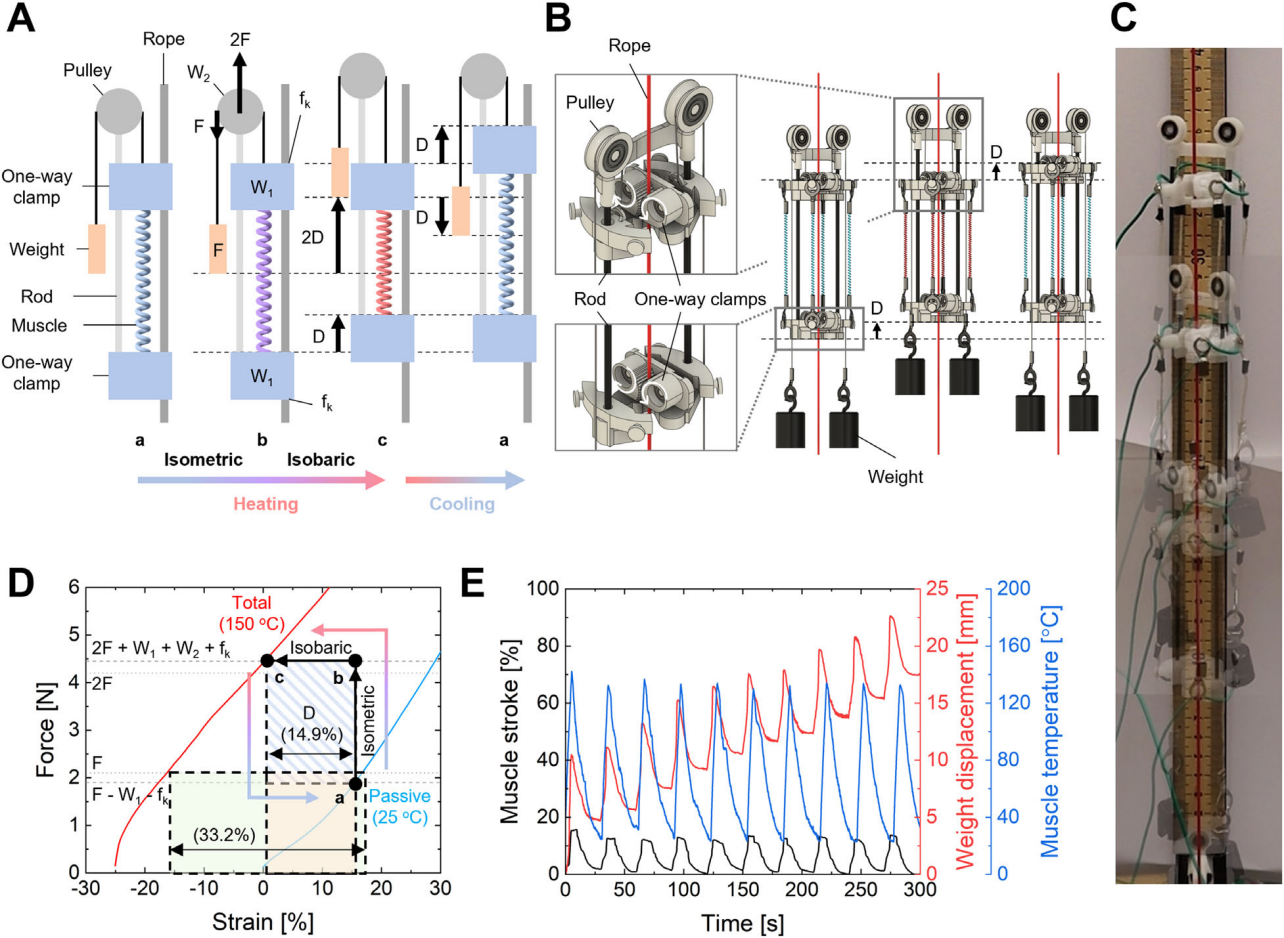
Work accumulation mechanism ‐ rope‐climbing robot. (A) Schematic of rope‐climbing robot. The rod is fixed to the bottom one‐way clamp and free to slide in the top one‐way clamp. The weight is connected to the top one‐way clamp through a string. (B) Schematic diagram of the robot working mechanism. The highlights show the rotation of the one‐way clamps. (C) Real‐time pictures showing the robot climbing up the rope. (D) Robot vertical movement prediction from the total and passive force curves. The total weight attached to the robot is 2.1 N, which is F in the plot. The muscles start from point a and are under the isometric condition before reaching point b. After reaching the threshold force in point b, the muscles start contracting and enter the isobaric condition. The blue box area is the robot energy input from the muscles for each cycle. The orange area is the robot work gained from the load vertical movement for each cycle. The green box is the work gained from muscle contraction under a traditional isobaric test. (E) Comparison of muscle stroke, weight vertical movement, and muscle temperature for ten cycles measured from the rope‐climbing robot.

To illustrate the benefits of supercoiled muscle, we develop a vertical rope‐climbing robot capable of lifting weights while climbing. Figure 7A outlines the working mechanism of the robot, which consists of two one‐way clamps, supercoiled muscle, rod, string, and pulley. The force from the hanging weight is F, the weight of the one‐way clamp is $W_{1}$, the combined weight of the pulley and rod is $W_{2}$, and the kinetic friction between the one‐way clamp and the rope is $f_{k}$. The specific arrangement realizes the general concept of Figure 6. The one‐way clamp is a custom device that uses an internal torsional spring mechanism to enable the rope to slide in one direction with friction lower than in the other direction. In our case, the ratio between directional friction forces is 84. A weight is then attached to a string connected to the upper one‐way clamp. Figure 7B,C provide schematics and real‐time images. Video S4 includes a video of the robot's working mechanism.

This robotic mechanism follows the general method to accumulate work from repeating cycles described in Figure 6. The cycle begins with step a to step b. During this stage, the muscles are heated up, but the force generated is insufficient to overcome the threshold force. This threshold force ($2F+W_{1}+W_{2}+f_{k}$) is shown in Figure 7D, which includes twice the weight due to the pulley mechanism, the weight of the one‐way clamp, the weights of the rods and pulleys, as well as the friction from the one‐way clamp. Hence, during this stage, the muscles are under an isometric condition. From step b to step c, the force reaches the threshold to induce motion in the allowable direction, and the bottom one‐way clamp opens, allowing sliding. During this step, the muscle contracts, and the force is constant, and hence, the muscle is under isobaric condition. From step c to step a, the muscles cool to room temperature. As the muscles cool, their active forces decrease until they reach the lower threshold force ($F-W_{1}-f_{k}$). The muscles then extend back to their length to step a due to the tension force exerted on the upper clamp through the string connected to the load. The width of the enclosed box in Figure 7D predicts the vertical movement of the robot after each cycle using the passive and total force curves. The predicted movement is 14.9%, which is similar to the stroke measurement shown in Figure 7E. Figure 7E illustrates the muscle stroke, temperature, and vertical movement of the robot over time.

The mechanism requires the following elements: two one‐way clamps, a pulley mechanism, and a weight hanging on the pulley to restore the tension of the muscles. We note that if we simply attach the weight to the lower clamp by a direct straight string without using the pulley, the robot would not be able to climb up because the muscles would not be able to restore their length during cooling. This is caused by the slenderness of the muscles, which prevents them from extending their length due to buckling if there is a compressive force. In this robot, the compressive force is from the friction force of the upper one‐way clamp. The combination of these elements is necessary to accumulate work within each cycle.

We note that in the traditional isobaric test, where the muscle lifts a load against gravity and lowers it back to the initial position upon cooling, the full‐cycle work is zero since the load returns to its original position. Consequently, most studies focus on comparing only the half‐cycle isobaric contractile work. The comparison between the robot full‐cycle work and the half‐cycle isobaric work is illustrated in Figure 7D. The blue shaded area represents the energy input from the muscles for each full cycle, which is 15.2 mJ. The orange area represents the potential energy gained from the weight's vertical movement for each full cycle, which is 12.5 mJ. The result demonstrates that the robot achieves an efficiency of 82.2%, meaning it converts 82.2% of the muscle's mechanical work into the weight's potential energy increase. To enhance efficiency, it is essential to minimize rope friction and reduce the weight of the robot's components. If we ignore the robot's weight and the friction force, the area of the orange box should be equal to the area of the blue box, which would represent 100% efficiency.

The green box illustrates the half‐cycle contractile work generated from muscle actuation during the traditional isobaric test, which is 27.9 mJ. The half‐cycle work produced by the robot is depicted in steps a to c, in which the muscles contract D and the load is elevated by 2D, resulting in a work output of 25 mJ. The difference in work between the half‐cycle isobaric test and the half‐cycle robot test can be attributed to the weight of the robot components and friction losses. After step c, the work decreases from step c to a due to the restoration of the muscle length in preparation for the subsequent cycle.

## 2 Discussion and Conclusion

We present hierarchical supercoiled muscles with J‐shaped passive force curve, inspired by biological muscle systems [21, 62]. Here, we measure the effect of the J‐shaped curve on the antagonistic arrangements that are essential for bio‐inspired robotic mechanisms, and on the work accumulation mechanisms, such as the rope climbing robot. Results obtained from the experimental validation of the use of hierarchical J‐curve muscles in antagonistic arrangements and work accumulation mechanism demonstrate enhancements of 75% and 480%, respectively, compared to coiled muscles with linear force‐displacement behavior. This highlights the target of this study, which is to establish the relation between antagonistic arrangements used in cyclic work and the J‐shaped nature of hierarchical muscles. These enhancements were obtained for the current supercoiled muscles, where the ratio between the passive strain and the active stroke, under the same tensile load, is larger than one. We expect notable improvements for J‐curved muscles, which have an even higher ratio of passive strain to active stroke, such as the hypercoiled muscles.

By adding an embedded wire in a super‐ or hypercoiled muscle, we also realize uniform heating and predictable actuation. Thermal actuation in twisted and coiled polymer actuators (TCPA) has so far presented a key challenge for their reliable use in soft robotics. Joule heating a resistive wire is the prevailing method due to its simplicity. There are two primary methods for implementing Joule heating: winding a heating wire around the muscle [53] and coating silver on the muscle [11, 12, 13, 14, 15, 56]. However, the former approach of winding a heating wire around the coils has limitations. If the wire is densely coiled, it adds weight and mechanical constraints, which reduce the work capacity. If the heating wire is not densely wrapped around the muscle fiber, it leads to inefficient heating with high heat losses to the ambient, heat concentration, or even localized melting. Some studies employ silver on polymer artificial muscles due to its excellent conductivity and resistance to oxidation. However, silver coating increases the tensile modulus [15], cannot withstand large strains, and cracks during the twist insertion. Our results show that the torsion and tension applied to the surface coating during manufacturing result in the formation of numerous microcracks and coating peel‐off during the process, as shown in Figure S8. The actuation of the coiled artificial muscle relies on the untwisting of the fiber cross‐section [10, 53, 58, 63, 64, 65], which results in greater radial strain near the outer surface of the fiber or bundle [57]. Due to this radial strain gradient, the placement of the heating wire near the center of the fiber bundle reduces the constraint on actuation compared to placement at the outer diameter. Moreover, we show in Figure S9, that the heating wire stiffness is negligible compared to the supercoiled muscle. Therefore, the supercoiled muscle design with an embedded heating wire in the center achieves a higher heating rate and better stroke performance.

Thanks to the high work capacity of supercoiled muscle, and most importantly, the J‐curve, our vertical rope‐climbing robot achieves a payload‐to‐body weight ratio of 14.6. This ratio is at least four times higher than that of robots powered by electric motors. For motor‐driven rope‐climbing robots, this ratio typically ranges from 1.3 to 3.7 [66, 67, 68, 69, 70, 71]. Pneumatic actuators are also commonly used for vertical rope‐climbing robots, often requiring three sets of actuators for inchworm‐inspired locomotion [57, 72, 73, 74, 75]. In this mechanism, the two actuators at both ends provide gripping and anchoring, while the central actuator extends the body to climb. Park et al. introduced a snap‐inflatable robot utilizing ratchets, reducing the number of actuators to one [76]. Xie et al. developed a fully 3D‐printed rope‐climbing robot with a payload‐to‐body weight ratio of 8 [72], whereas the snake‐inspired robot achieved a ratio of 25 [73] without considering the huge mass of the bulky external pump.

We developed a first‐principles computational model of twisted and coiled polymer actuators (TCPAs) based on Cosserat rod theory. In this study, we use this model to analyze muscle behavior in both passive and active states, uncover the physical origins of the nonlinear passive J‐curve characteristic of supercoiled muscles, and predict the equilibrium configuration of antagonistic muscle pairs within an elbow‐joint mechanism. Unlike previous data‐driven approaches or reduced‐order linear helical‐spring models, our framework predicts both active and passive actuator behavior directly from the material properties (including friction), geometry, and topology of the individual fibers. This capability opens new opportunities for computational performance optimization of TCPAs and for their integration into multi‐muscle, articulated devices. A key design variable not captured by earlier simplified models is the interfacial interaction between fibers, which we demonstrate to be a dominant contributor to the J‐curve nonlinearity. Other important design parameters that remain unexplored in prior work include the geometry of individual fibers, fiber‐twist chirality, the number of fibers, and the topology of fiber bundles. This broader design space –largely unexamined in the supercoiled polymer actuator literature– has potential to yield advantageous nonlinear behaviors, as shown in this paper. Future work will use our computational framework to systematically explore this rich material design possibility.

Inspired by the hierarchical structure of human skeletal muscle, we develop an innovative polymer‐based supercoiled artificial muscle featuring a non‐linear stress–strain curve, which shows benefits in muscle antagonistic arrangement and work accumulation mechanism. We also predict the strain of antagonistic muscle pairs based on their total and passive force curves and the Cosserat rod simulation. Traditional monofilament twisted and coiled artificial muscles require wrapping an external heating wire for Joule heating actuation, which leads to a temperature standard deviation of 18.7 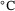. In contrast, Supercoiled muscles offer a more uniform temperature distribution since heat is generated from the center of the muscle, resulting in a much lower temperature standard deviation of 2.5 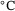. With the temperature uniformity and the stroke consistency of supercoiled muscle, we construct a heat transfer model to predict muscle temperature, allowing the estimation of muscle stroke. With its combined advantages, the supercoiled muscle opens new possibilities in soft robotics, providing improved and repeatable performance for a wide range of soft actuator applications.

## 3 Experimental Section

### 3.1 Muscle Fabrication Process

The nylon fibers were purchased from RUNCL (PowerMono fishing line 0.47 mm). Before fabrication, three nylon fibers and a heating wire were anchored at the top end, with the lower ends attached to weights. All the fibers and heating wire pass through different branches of the channel. The fabrication involved two motors: a rotary motor that controls the top end with the fibers and wire and a linear motor connected to the channel. As the rotary motor initiated the plying process, the linear motor simultaneously moves downward. After plying the fibers and the wire, the three‐ply fiber was twisted once more before the onset of self‐coiling to store additional elastic energy. The twisted three‐ply fiber was then coiled around a mandrel to create its spring‐like geometry, with the final shape fixed through annealing at 180 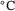 for 8 h. Hypercoiled muscle followed a similar process to the supercoiled muscle. The initial step involved plying three monofilament fibers to form a three‐ply fiber. Three such three‐ply fibers were subsequently piled before twisting and coiling. Table 1 compares the muscle parameters. Note that all three muscles had a similar total cross‐sectional area. Additionally, these muscles exhibit a comparable spring index as a result of using the same mandrel during the coiling step. A detailed description of the fabrication process can be found in Figure S10 and Video S1.

The high frequency supercoiled muscles were made from 50 μ m nylon fiber (STROFT Fishing Line). Similar to the previous method, three nylon fibers were plied and twisted together first, but without the electrical heating wire. 0.2 mm Nichrome wire was used as a mandrel during the coiling process. After one hour of annealing in a vacuum oven at 170 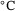, the first mandrel was replaced with 0.15 mm Nichrome wire. The muscle was stretched to provide enough gaps between coils, and annealed again in the vacuum oven for another hour at the same temperature. To enable Joule heating, we applied a thin layer of CNT at the surface of the muscle by winding a continuous CNT strip from the CNT forest. Then, a few droplets of IPA were applied to the muscle to make sure the CNT layer was well fixed to the muscle. The muscle was connected to the electrical wires using silver paint (Ted Pella, Inc.) and high heat resistant epoxy (J‐B Weld) before testing with DMA. We used the scanning electron microscope (SEM, Hitachi S‐4800) to ensure the surface was fully covered by CNT, which imparts electrical conductivity.

### 3.2 Muscle Isobaric Test

During the isobaric test, one end of the muscle was fixed, and the other end was connected to the linear guide. The weight was connected to the linear guide by a string, and the force of the weight was redirected by two pulleys. Both ends of the muscle were tethered to ensure no end rotation. The heating wire was connected to the power supply. Here, we used NI DAQ (USB‐6210) to control the power supply and fan for active cooling. The displacement of the muscle was measured with a laser sensor, and the muscle temperature was measured with an IR camera (FLIR A700) or thermocouple. All data was collected by NI DAQ. The testing setup is shown in Figure S11.

The miniature high‐frequency muscles were lightweight and sensitive to weight. Thus, we tested them with DMA for precise results. We applied 29 V and 27 mA current to the muscle using the power supply. The two different working frequencies were tested (0.5 and 1 Hz) with a duty cycle of 50%.

### 3.3 Heat Transfer Simulation

For the COMSOL Multiphysics simulation, both the coiled and supercoiled muscle models possess identical polymer volume and helical geometry, and they had the same heating wire length and diameter. In the model, the muscle was heated by passing an electric current through the heating wire. Note that in both models, the muscle and heating wire were enclosed within a large air box to account for convection and conduction. In the coiled muscle model, there was a small air gap between the heating wire and the nylon fiber to replicate the experimental observation. In contrast, the supercoiled muscle model features the heating wire embedded in the center of the nylon fiber, enabling conduction between the surface of the heating wire and the surrounding nylon fiber. The fitting parameter for the model was the convection heat transfer coefficient, which was set to be 50 $Wm^{-2}K^{-1}$ for both coiled and supercoiled muscle.

### 3.4 Muscle Dynamics Computational Modeling ‐ Cosserat Rod

#### 3.4.1 Muscle Geometry Parametrization

Let {i,j,k} be an orthonormal basis for the 3D global frame. The parametrization of the geometries of the coiled and supercoiled muscles was derived analytically in this section.

#### 3.4.2 Coiled Muscle Geometry

The coiled muscle was modeled as an ideal helix. The centerline position of a coiled muscle $x_{m}$ shown in Figure 4A of length $L_{m}$ with coil radius $R_{m}$ and number of coils per muscle length $k_{m}$ can be parameterized as follows:

(1) $$x_{m}\left(s\right)=R_{m}cos\left(k_{m}s\right)i+R_{m}sin\left(k_{m}s\right)j+sk,\;s\in \left[0,L_{m}\right]$$

#### 3.4.3 Supercoiled Muscle Geometry

Each supercoiled fiber took the shape of a helix wrapped around a helix as shown in Figure 4B. The centerline position of this shape could be derived by first parameterizing the center helix each fiber wraps around, then defining local normal and binormal vectors to parameterize another helix around the center helix. The geometry of the center helix $x_{c}$ for a supercoiled muscle of length $L_{s}$, coil radius $R_{s}$, and number of coils per muscle length $k_{s}$, can be described in the same way as the coiled muscle:

(2) $$x_{c}\left(s\right)=R_{s}cos\left(k_{s}s\right)i+R_{s}sin\left(k_{s}s\right)j+sk,\;s\in \left[0,L_{s}\right]$$

Differentiating this expression for $x_{c}$ with respect to s, gives the tangent vector t:

(3) $$t\left(s\right)=\frac{dx_{c}}{ds}\left(s\right)=-R_{s}k_{s}sin\left(k_{s}s\right)i+R_{s}k_{s}cos\left(k_{s}s\right)j+k,\;s\in \left[0,L_{s}\right]$$

Differentiating the tangent vector and normalizing gives a unit normal vector N:

(4) $$N\left(s\right)=\frac{\frac{dt}{ds}\left(s\right)}{\left\|\frac{dt}{ds}\left(s\right)\right\|}=-cos\left(k_{s}s\right)i-sin\left(k_{s}s\right)j,\;s\in \left[0,L_{s}\right]$$

The binormal vector B can be defined by normalizing the tangent vector and taking the cross product with the normal vector:

(5) $$\begin{array}{ccc} B(s) & = & \frac{t(s)}{∥t(s)∥}\times N\left(s\right)=\frac{1}{\sqrt{R_{s}^{2}+1^{2}}}\left(sin\left(k_{s}s\right)i-cos\left(k_{s}s\right)j+R_{s}k\right),\\ & & \;s\in [0,L_{s}] \end{array}$$

Now, the geometry of a supercoiled muscle fiber $x_{s}$ that wraps around the center helix $k_{p}$ times per muscle length at a radius of $R_{p}$ can be parametrized as follows:

(6) $$\begin{array}{ccc} x_{s}\left(s\right) & = & x_{c}\left(s\right)+R_{p}cos\left(k_{p}s+\theta \right)N\left(s\right)+R_{p}sin\left(k_{p}s+\theta \right)B\left(s\right),\\ & & \;s\in [0,L_{s}] \end{array}$$

here, θ is used to offset the fibers around the center helix $x_{c}$. For a supercoiled muscle with three fibers, θ will take the values 0,2π/3, and 4π/3 for each fiber, respectively.

#### 3.4.4 Muscle Fiber Dynamics

Each muscle fiber was modeled as a Cosserat rod. The Cosserat rod model shown in Figure 4C was a mathematical model of 3D motions and deformations of 1D slender elastic bodies. This model presented several advantages for modeling the coiled artificial muscles: (I) it captured all modes of deformation (bending, twist, shear, and stretch), prevalent in elastomeric materials; (II) kinematic or dynamic boundary conditions could be directly enforced, allowing the assembly of multiple rods into complex layouts, such as the supercoiled muscle; (III) contact dynamics and friction could be easily incorporated into the Cosserat rod dynamics as external forces and torques [35, 38, 50], making it easier to model fiber‐fiber interactions in the supercoiled muscle.

#### 3.4.5 Cosserat Rod Theory

A Cosserat rod of length L(t) at time t was described at each center line arc length coordinate s∈[0,L(t)] by a position vector $x\left(s,t\right)\in R^{3}$ and an orthonormal frame $Q\left(s,t\right)\in R^{3\times 3}=\left\{d_{1},d_{2},d_{3}\right\}$ (triad of unit vectors), which defines the frame transformation between the global and local frames. Any vector $v\in R^{3}$ defined in the global frame can be transformed into a local frame counterpart $\bar{v}\in R^{3}$ by $\bar{v}=Qv$ and back $v=Q^{T}\bar{v}$. For an unshearable and inextensible rod, $d_{3}$ was always parallel to the rod tangent t, with $d_{1}$ (normal) and $d_{2}$ (binormal) spanning the rod cross‐section. Extension and compression could be captured by letting the current rod arc‐length s differ from the reference rod arc‐length $\hat{s}$, and was quantified using the dilatation field $e=ds/d\hat{s}$. To make the rod shearable, we allowed $d_{3}$ to detach from the tangent t and the shear σ can be quantified by the difference between the two $\sigma =d_{3}-et$. We define the curvature vector $\kappa \left(s,t\right)\in R^{3}$ through

(7) $$\frac{\partial d_{i}}{\partial s}=\kappa \times d_{i}$$

where it is used to encode the rotations of the local frames Q across the length of the rod. We denote velocity by $v\in R^{3}$, angular velocity by $\omega \in R^{3}$, second inertial area moment by $I\in R^{3\times 3}$, density by ρ, and cross‐sectional area by A. We assume a constant circular cross‐section for all the muscles in this study. Finally, the dynamics of the rod can be described using the following set of equations:

(8) $$\frac{\partial x}{\partial t}=v$$

(9) $$\frac{\partial d_{i}}{\partial t}=\omega \times d_{i}$$

(10) $$\frac{\partial^{2}\left(\rho Ax\right)}{\partial t^{2}}=\frac{\partial n}{\partial s}+f$$

(11) $$\frac{\partial (\rho \bar{I}\bar{\omega })}{\partial t}=\frac{\partial \bar{\tau }}{\partial s}+\bar{\kappa }\times \bar{\tau }+Qt\times \bar{n}+\left(\rho \bar{I}\bar{\omega }\right)\times \bar{\omega }+\bar{c}$$

where the last two equations represent the linear and angular momentum balance at each cross section, n and τ are the internal forces and torques, f and c are the external force and torque densities, respectively. It is necessary to specify the form of the internal forces n and torques τ generated in response to bending/twisting strains κ, and shearing/stretching strains σ. We assume a perfectly elastic material so that the stress–strain relations can be expressed as

(12) $$n=S(\sigma -\sigma_{0})$$

(13) $$\tau =B(\kappa -\kappa_{0})$$

We introduce the intrinsic shear strain $\sigma_{0}\in R^{3}$, and intrinsic bending curvature $\kappa_{0}\in R^{3}$ to allow the rod to take a non‐straight rest configuration. Furthermore, the shear matrix is denoted by $S\in R^{3\times 3}$ and the bending matrix by $B\in R^{3\times 3}$. Denoting the radius of the muscle fiber as r, its Young's modulus as E, and shear modulus as G we can express the shear and bending matrices more explicitly as:

(14) $$S=\pi Er^{2}\left[\begin{array}{ccc} 4G/3E & 0 & 0\\ 0 & 4G/3E & 0\\ 0 & 0 & 1 \end{array}\right]$$

(15) $$B=\frac{\pi Er^{4}}{4}\left[\begin{array}{ccc} 1 & 0 & 0\\ 0 & 1 & 0\\ 0 & 0 & 2G/E \end{array}\right]$$

The matrices are written this way to emphasize how they scale with Young's modulus E and fiber radius r, which will become relevant when thermal actuation is discussed.

#### 3.4.6 Discretization and Numerics

The muscle fiber centerlines were discretized into $n_{elem}+1$ nodes of position $x_{i}$ connected by $n_{elem}$ cylindrical elements. The number of elements was chosen so that it could adequately capture the geometry and topology of each muscle fiber, as well as ensuring results were converged. Rod displacements were determined by the internal and external forces acting at the nodes, while rotations were determined by the internal and external torques applied to the cylindrical elements. The muscle fiber dynamics were computed by using a second‐order position Verlet scheme to integrate (with respect to time) a discretized version of the dynamics equations with appropriate boundary conditions [35]. This numerical approach had been validated against a number of benchmark problems with known analytic solutions, as well as experimental investigations involving contact and anisotropic surface friction [35, 39, 42, 43]. In this study, we used PyElastica [34], which is the Python‐based implementation of this numerical scheme Elastica.

#### 3.4.7 Muscle Fiber Topology

Parameterizing the geometry of the muscle fibers was not enough to capture the twist of the muscle fibers. This twist could be captured in the model by rotating the local frames Q at each arc length coordinate s around the tangent vector T as shown in Figure 4D. We used concepts from knot theory to determine the amount of twist that needs to be inserted into the muscle fibers using this method. Specifically, the link (Lk), the writhe (Wr), and the twist (Tw) were used to quantify the inserted fiber twist. The link (Lk), was a topological invariant defined as the oriented crossing number (or Gauss linking integral) of the centerline and one of its edges (the green auxiliary line) averaged over all projections [77]. The writhe was a global geometric quantity that captures how much a curve bends and coils, but did not account for the orientation of the edges [78]. Twist (Tw), was another geometric quantity that measures the total rotation of the auxiliary green curve about the centerline's tangent. These three quantities were related through the Calugareanu‐Fuller‐White (CFW) theorem [78, 79], which states that:

(16) Lk=Wr+Tw

During the muscle manufacturing process, each muscle fiber was twisted and coiled a known number of times, which translated into a known link value $Lk_{m}$ for each muscle fiber. In addition, parameterization of the geometry of the muscle fiber could be used to compute the writhe $Wr_{c}$ and twist $Tw_{c}$ of the muscle fiber without inserted twist [37, 80]. To satisfy the (CFW) theorem, the injected twist ΔTw must be equal to the difference between the sum of the computed twist and writhe and the measured link value:

(17) $$\Delta Tw=Lk_{m}-\left(Wr_{c}+Tw_{c}\right)$$

Then, the frames of the Cosserat rod element $Q_{i}$ are linearly rotated around the respective tangent $t_{i}$ such that the first frame is not rotated at all and the frame at the end is rotated by 2πΔTw.

#### 3.4.8 Muscle Fiber Thermal Actuation Model

The nylon polymer muscle fibers expanded anisotropically when they were heated, causing them to untwist and contract the muscle by ΔL [53]. In our model, we incorporated the thermal effects on muscle fibers by allowing the fiber radius r and Young's modulus E to vary with temperature T(t), which was a function of time. This in turn affects both the shear S and the bending B matrices and makes them functions of temperature T, which we account for by modifying the stress–strain relations as follows:

(18) $$n=S\sigma -S_{0}\sigma_{0}$$

(19) $$\tau =B\kappa -B_{0}\kappa_{0}$$

where $S_{0}$ and $B_{0}$ are the initial shear and bending matrices, respectively. These new relations would result in internal forces n and torques τ in the muscle fibers in response to changes in the bending and shear matrices over time. Since these matrices change only with the fiber radius r and Young's modulus E, the decrease in Young's modulus E and increase in fiber radius r with increasing temperature were what cause the overall fiber untwisting shown in Figure 4E. We assumed that the fiber radius r undergoes linear thermal expansion with a thermal expansion coefficient α. The change in Young's modulus with temperature for the coiled nylon fiber was more difficult to measure, and it had a more significant effect on the actuation than the fiber radius, so we cannot assumed that it was constant or changes linearly with temperature. To quantify the change in Young's modulus with temperature, we introduce the dimensionless quantity ϕ:

(20) $$\phi =\frac{Er^{4}}{E_{0}r_{0}^{4}}$$

where $E_{0}$ and $r_{0}$ are the initial Young's modulus and the fiber radius, respectively. This quantity encodes the change in the bending matrix with time through the following relation,

(21) $$B=\phi B_{0}$$

The muscle contraction ΔL is mainly caused by the untwist of the muscle fibers resulting from the internal torque τ along $d_{3}$ due to the change in B with temperature in Equation (19). Since ϕ controls the change in B, it follows that the contraction amount ΔL is controlled by ϕ. Unlike E, ΔL can be measured easily and directly with changing temperature. Therefore, it is more convenient to fit ϕ to match the experimental contraction value ΔL with temperature to compute E than to experimentally measure the Young's modulus of the coiled fiber with temperature. This is done by assuming that $\phi =1+c{\left(\Delta T\right)}^{n}$ and tuning c and n to match the experimental contraction ΔL with temperature. The tuned actuation parameters are shown in Table S1.

#### 3.4.9 Fiber–Fiber Interactions

To accurately model the supercoiled muscles, the interactions between the supercoiled muscle fibers must be included in the Cosserat rod model. These interactions include contact, friction, adhesion, and sliding.

#### 3.4.10 Contact Model

We employed the Hertzian contact model [55] shown in Figure 4F, to model the contact between the curved surfaces of the muscle fibers. This contact force could be modeled as a repulsive force $F_{N}$ between two rod elements i and j proportional to the normal penetration $\varepsilon_{N}=2r-\left\|d_{i}\right\|$ along the direction of the unit vector $u_{i}=d_{i}/\left\|d_{i}\right\|$ between the two element centers $d_{i}={\bar{x}}_{j}-{\bar{x}}_{i}$. The force on element i can be expressed as

(22) $$F_{N,i}=-H\left(\varepsilon_{N}\right)k_{n}\varepsilon_{N}^{3/2}u_{i}$$

where $H(\varepsilon_{N})$ is a Heaviside function that ensures that a force is applied only when there is penetration ($\varepsilon_{N}\geq 0$), and $k_{n}$ is the contact stiffness coefficient, which is a function of the material and geometric properties and is tuned to fit the experimental results. We applied this force between pairs of elements that were near each other, as in immediate neighboring elements in separate fibers, as well as the elements directly adjacent to them to capture the effects of sliding.

#### 3.4.11 Adhesion Model

To capture the passive nonlinear J‐curve like response of the supercoiled muscles, a linear spring model shown in Figure 4G is implemented to model the adhesion between the supercoiled muscle fibers. This adhesion model was applied to each pair of fibers in the supercoiled muscle.

The spring connects the surface of one fiber element to the surface of the nearest element in a second fiber. Here, the connections were initially defined based on the nearest surface points between fibers in their initial configuration. However, as fibers rotate and deform during stretching/actuation, these points may separate. The connection model aims to generate forces and torques to align these points and minimize their separation. This spring model was based on a model for the connection between biological muscle fibers in octopus arms [38]. The spring model was defined as follows, given the two elements, one from each fiber, $R_{i}$ for fiber i∈{1,2}, we defined a force‐displacement boundary condition proportional to the distance between two points on the discretized surface of each rod ${\bar{x}}_{i}^{s}={\bar{x}}_{i}^{c}+r_{i}{\bar{s}}_{i}$, where ${\bar{x}}_{i}^{c}$ denotes the element center position of $R_{i}$, $r_{i}$ is the element's radius and ${\bar{s}}_{i}$ is the unit vector pointing from ${\bar{x}}_{i}^{c}$ to the ${\bar{x}}_{i}^{s}$. The unit vector was defined in the rest configuration such that ${\bar{s}}_{1}$ points from ${\bar{x}}_{i}^{c}$ to ${\bar{x}}_{j}^{c}$ and ${\bar{s}}_{1}=-{\bar{s}}_{2}$. At each timestep, a restoring force was applied based on the distance between these two points $\delta_{c,i}=\left\|d_{i}^{s}\right\|=\left\|{\bar{x}}_{j}^{s}-{\bar{x}}_{i}^{s}\right\|$ for i∈{1,2} and j∈{2,1}. Additionally, these restoring forces result in restoring torques with lever arm of $r_{i}{\bar{s}}_{i}$ that preserved the relative orientation between the elements as shown in Figure 4G. The force response $F_{c}$ for all the springs with respect to the distance vector $d_{i}^{s}$ can be described as

(23) $$F_{c,i}=k_{c}d_{i}^{s}$$

where $k_{c}$ is a fitting parameter based on experimental data, they are reported in Table S2.

Consequently, the restoring torques applied to each element can be determined using the following

(24) $${\bar{C}}_{i}=r_{i}{\bar{s}}_{i}\times {\bar{F}}_{c,i}$$

When the muscle was stretched, the muscle fibers began to slide past each other. This sliding caused the elements to rearrange relative to each other, which increased the contact force as a result of the elements pressing more on the neighboring elements. The net result of these contact force changes was an overall non‐linear J‐curve like the response from the supercoiled muscle.

## Funding

This work was supported by the Office of Naval Research (N00014–22–1–2569).

## Conflicts of Interest

The authors declare no conflicts of interest.

## Supporting information

Supporting Information

Supplemental Video 1

Supplemental Video 2

Supplemental Video 3

Supplemental Video 4

## Data Availability

All data needed to support the conclusions of this manuscript are included in the main text or the Supplementary Materials. Computational modeling was performed using PyElastica, which is publicly available at www.cosseratrods.org. Code and scripts associated with the coiled artificial muscles are available upon request.
