# Supplementary material for: Hierarchical Artificial Muscle with Nonlinear Elasticity for Antagonistic and Cyclic Robotics
Source: Adv Sci (Weinh). 2026 Mar 19;13(30):e21604. doi: 10.1002/advs.202521604 (PMC13248829; doi:10.1002/advs.202521604)
Supplement: Supplementary file 1 — Supporting Information [file ADVS-13-e21604-s005.pdf]

Supplementary Materials for Hierarchical Artificial Muscle with Nonlinear Elasticity for Antagonistic and Cyclic Robotics

Samuel Tsai[1], *Liuyang Cheng*[1], *Ali Albazroun*[1], *Qiong Wang*[1], *Jeongmin Kim*[1], *Arman Tekinalp*[1], *Soonwook Kim*[1], *Charlie Simcox*[1], *Ryne Downing*[1], *Vagish Sivaramakrishnan*[1], *Grace Carsello*[1], *Miles Bimrose*[1], *Wonsik Eom*[1], *William P. King*[1], *Mattia Gazzola*[1], and *Sameh Tawfick*[1*],

[1]Department of Mechanical Science and Engineering, Grainger College of Engineering, University of Illinois Urbana-Champaign, Urbana, IL 61801, USA.
[*]Corresponding author. Email: tawfick@illinois.edu

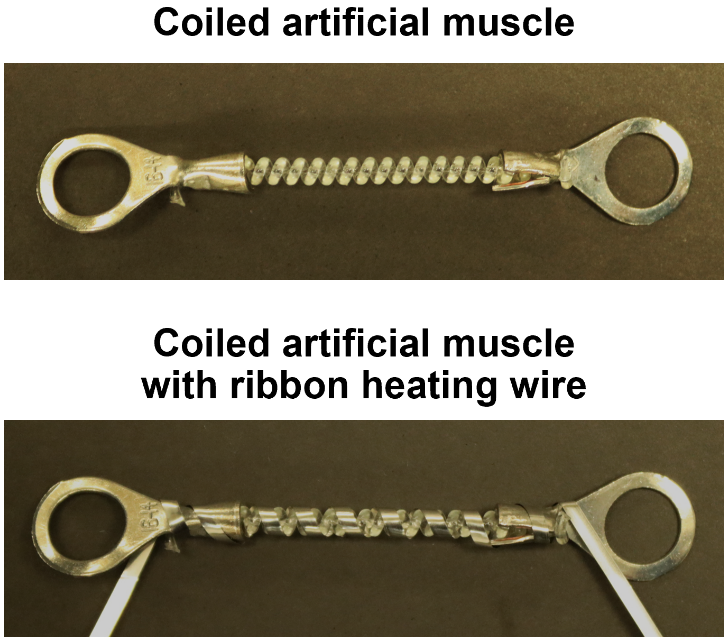

Figure S1: Coiled muscle wrapped with ribbon heating wire

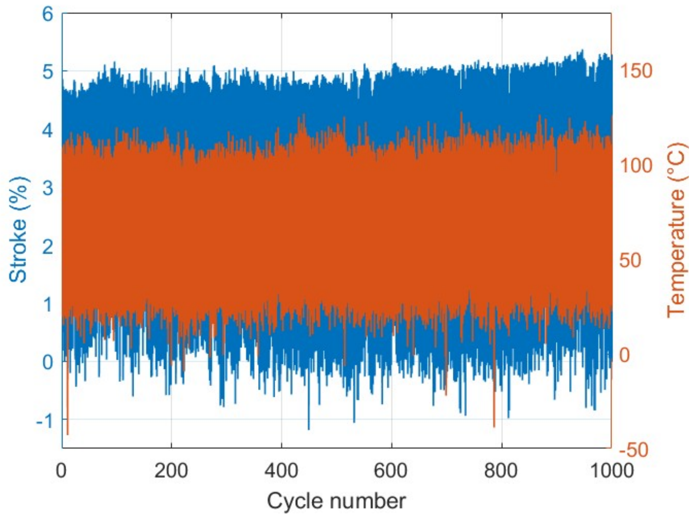

Figure S2: Hypercoiled muscle 1k-cycle test. Each cycle is 15 s with 5 s heating and 10 s cooling. The load is 85 g.

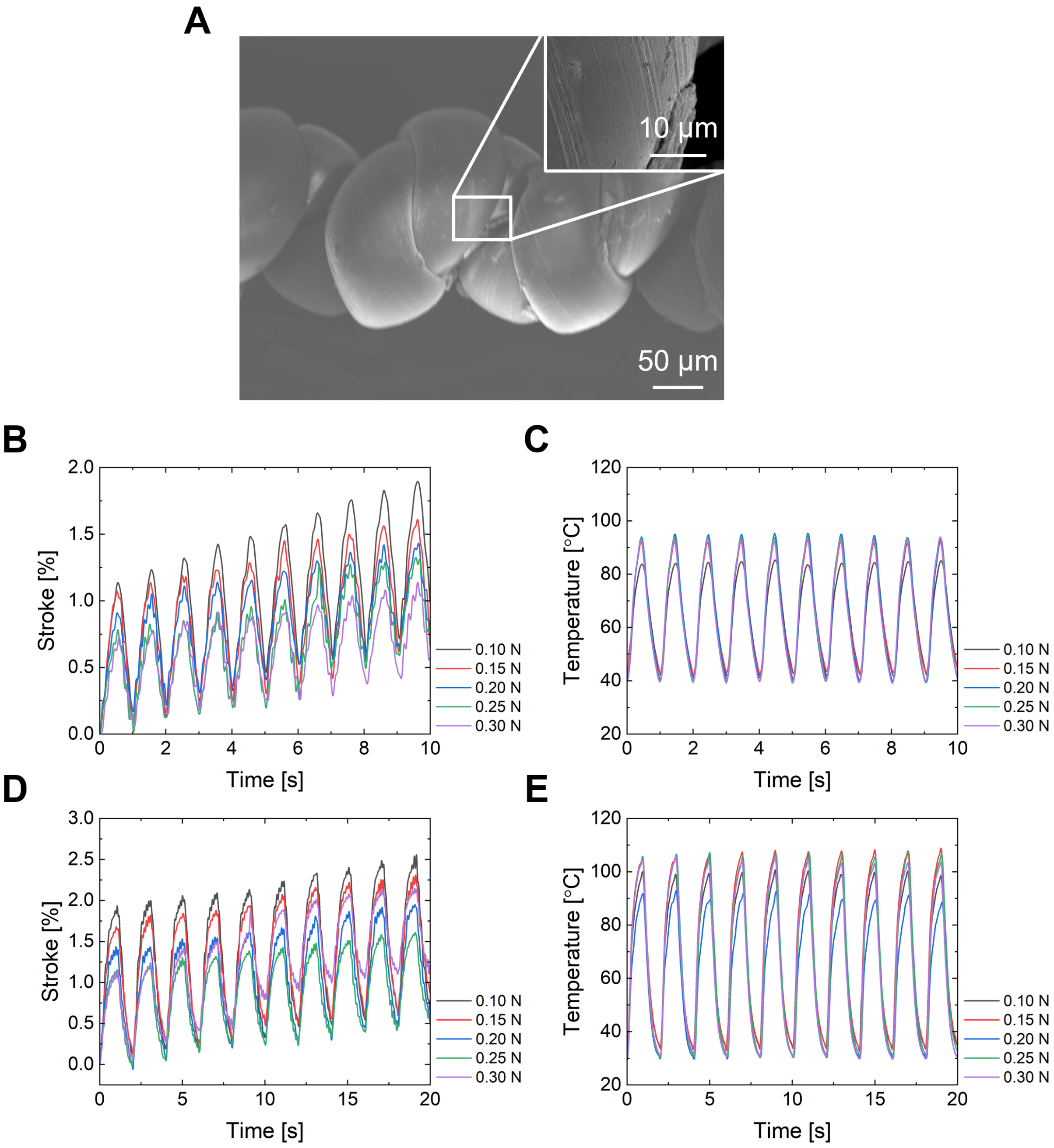

Figure S3: (**A**) 50 um mandrel supercoiled muscle without CNT coating. (**B, C**) Stroke and temperature change at 1 Hz. (**D, E**) Stroke and temperature change at 0.5 Hz.

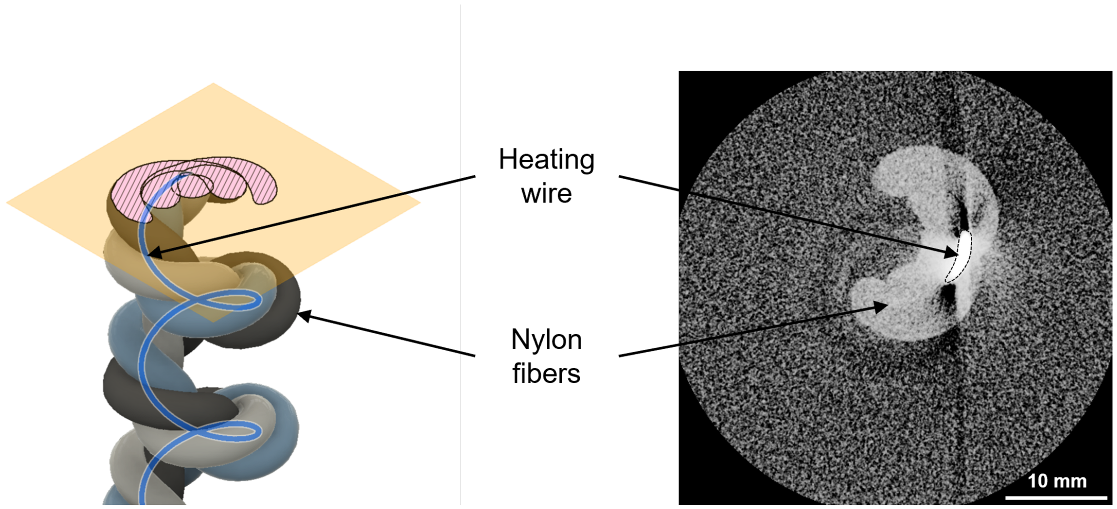

Figure S4: Supercoiled muscle computed tomography (CT) scan result

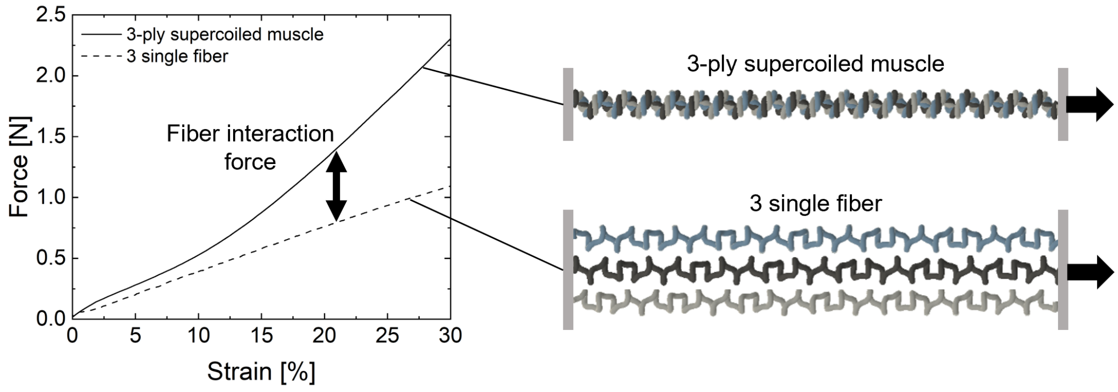

Figure S5: Tensile test for supercoiled muscle and individual fiber

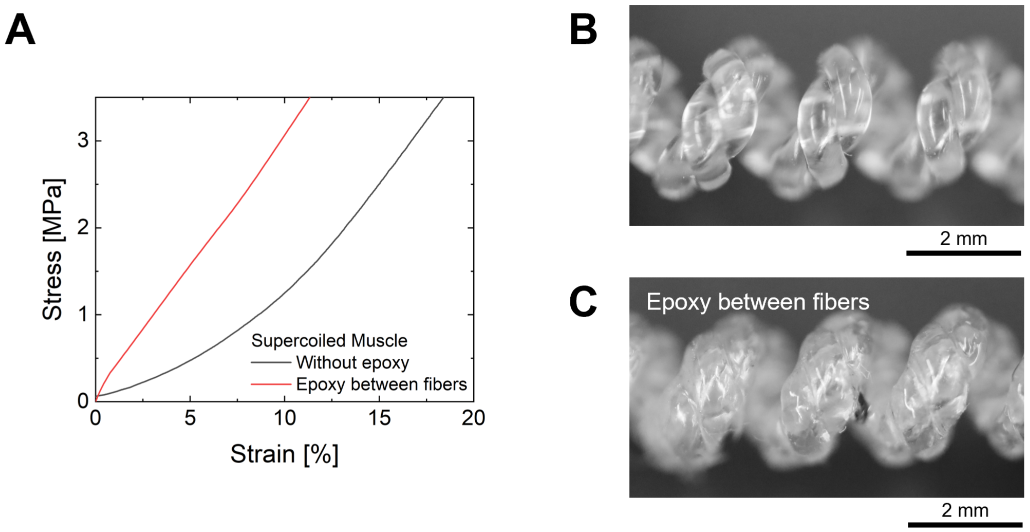

Figure S6: Supercoiled muscle with epoxy between fibers

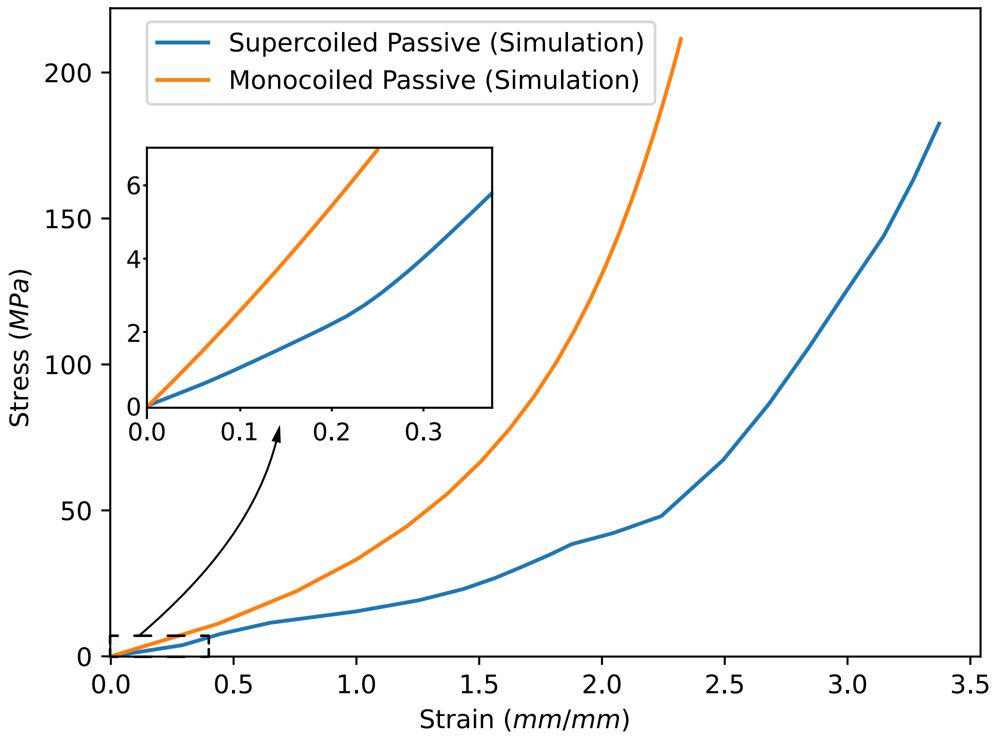

Figure S7: Passive curve for supercoiled muscle and coiled muscle to high strain

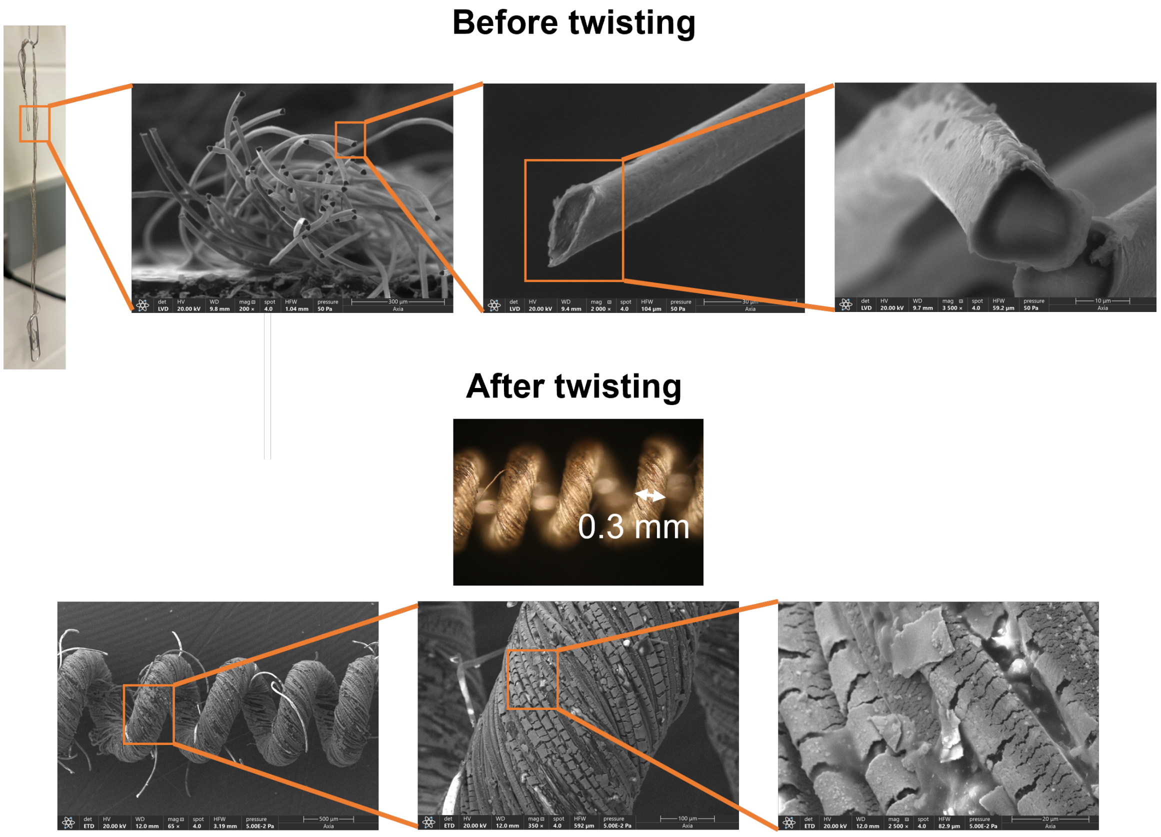

Figure S8: Coiled muscle made with coated nylon fibers

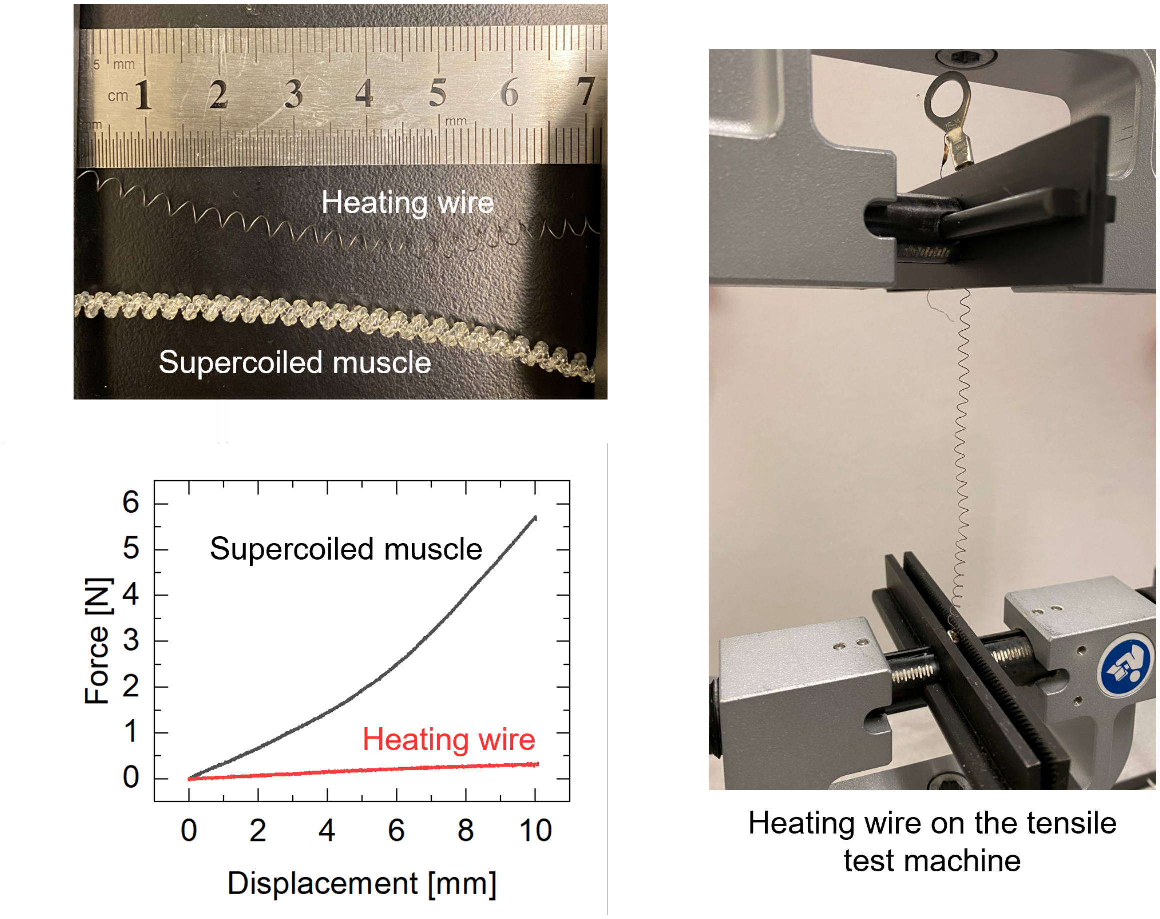

Figure S9: Stiffness of heating wire

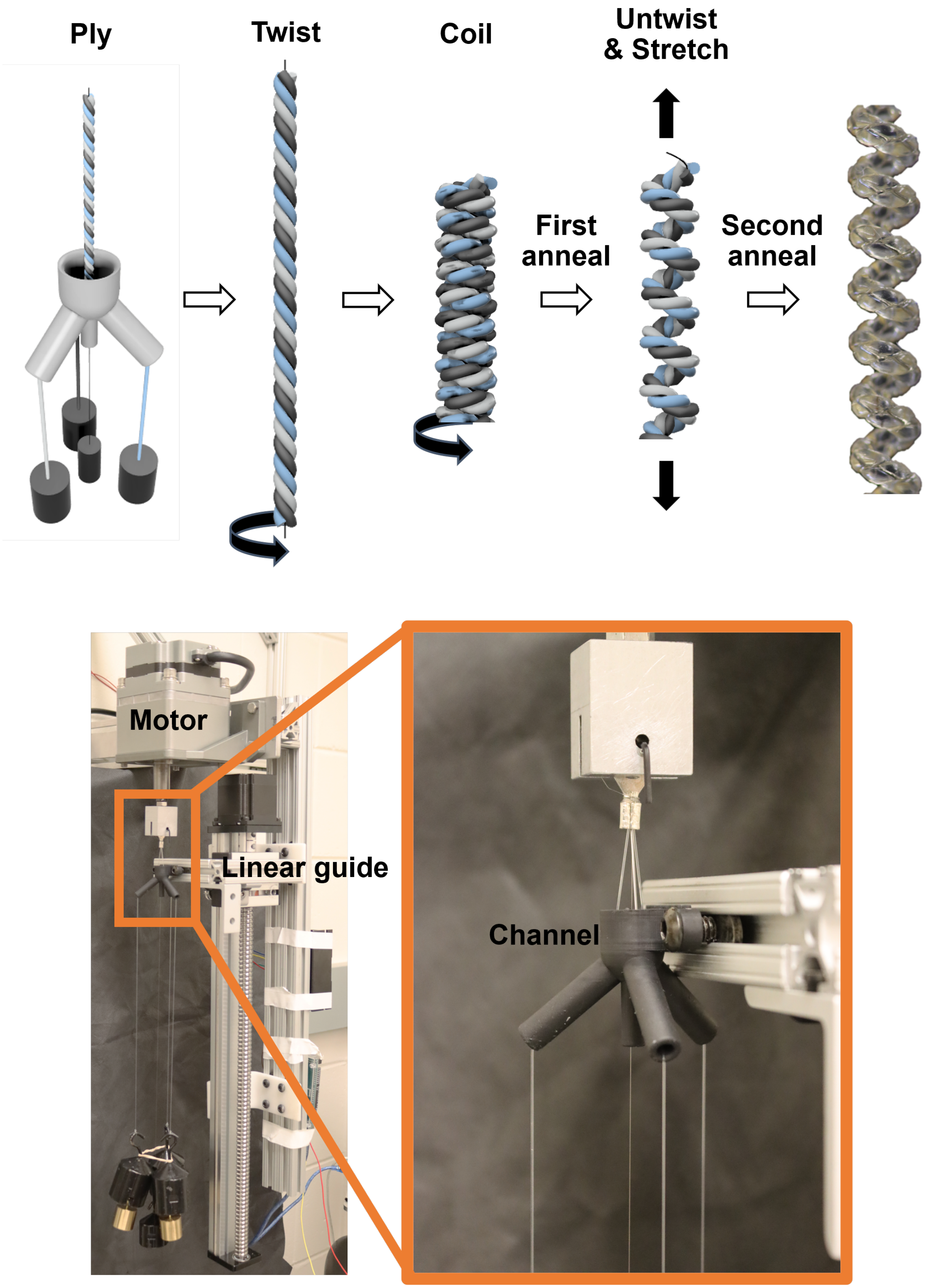

Figure S10: Fabrication process

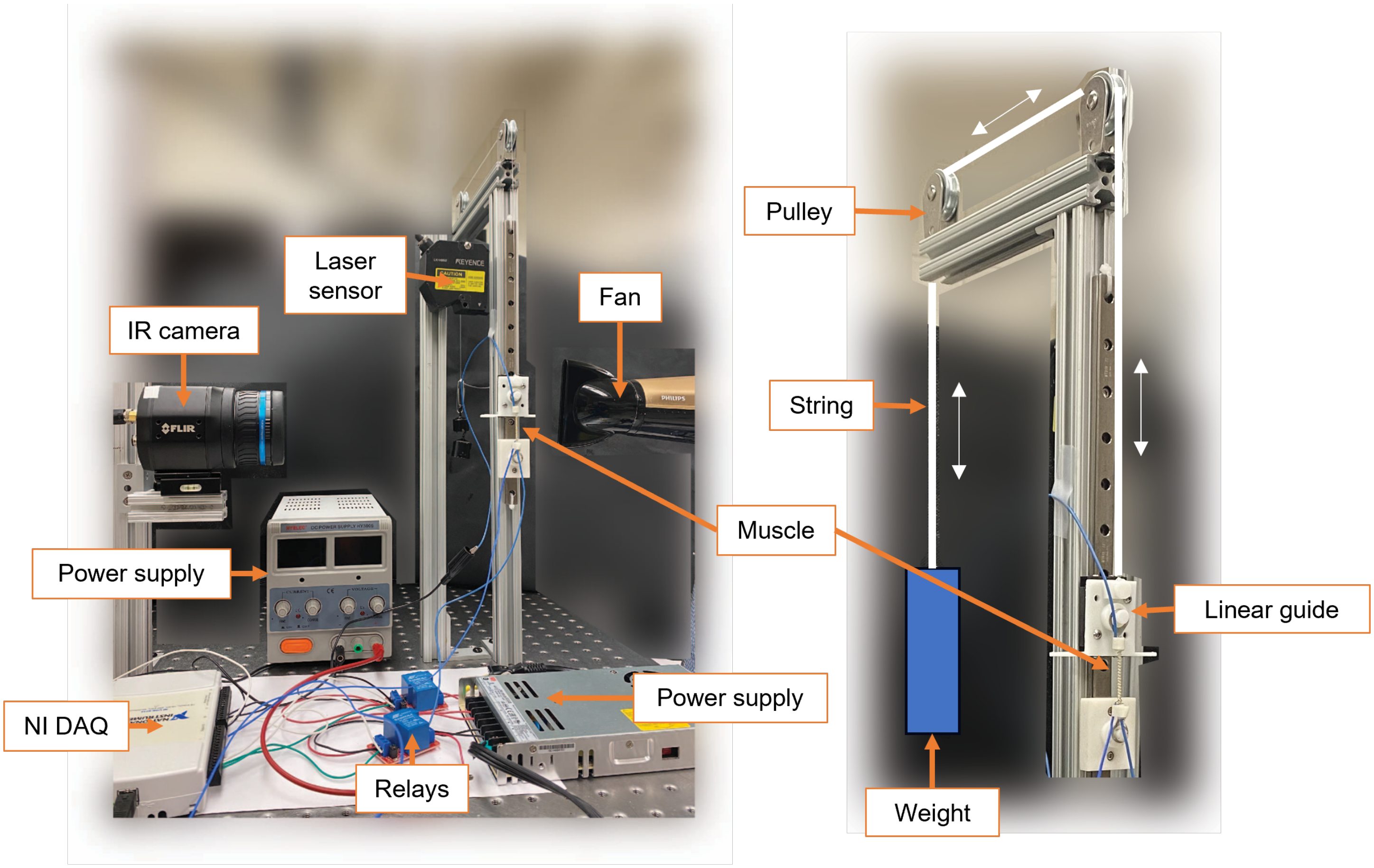

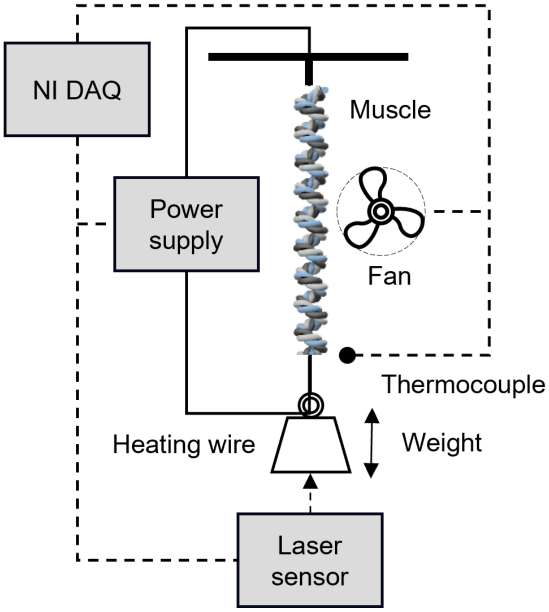

Figure S11: Isobaric testing setup and schematic

Table S1: Parameters for the thermal actuation model.

Parameter	Coiled Value	Supercoiled Value
n	0.85	2.6
c	6.38×10^{-4}	5.40×10^{-7}

Table S2: Fitting parameters for adhesion and contact model in Fig. 4F and 4G.

Parameter	Value (mN/m)
k_c	5.30
k_n	3.54

Movie S1: Supercoiled muscle fabrication video. The complete fabrication process of supercoiled muscle in Fig. 1C.

Movie S2: Actuation video of supercoiled muscle. The full cycle actuation of supercoiled muscle in Fig. 2B.

Movie S3: Supercoiled muscle antagonistic arrangement. The video version of Fig. 5A.

Movie S4: Rope-climbing robot. The video version of Fig. 7C.
